# Prediction of Complex Traits: Robust Alternatives to Best Linear Unbiased Prediction

**DOI:** 10.3389/fgene.2018.00195

**Published:** 2018-06-05

**Authors:** Daniel Gianola, Alessio Cecchinato, Hugo Naya, Chris-Carolin Schön

**Affiliations:** ^1^Department of Animal Sciences, University of Wisconsin-Madison, Madison, WI, United States; ^2^Department of Dairy Science, University of Wisconsin-Madison, Madison, WI, United States; ^3^Department of Plant Sciences, TUM School of Life Sciences, Technical University of Munich, Munich, Germany; ^4^Department of Agronomy, Food Natural Resources, Animals and Environment, Università degli Studi di Padova, Padova, Italy; ^5^Institut Pasteur de Montevideo, Montevideo, Uruguay

**Keywords:** complex traits, prediction, genomic selection, quantitative genetics, genome-enabled prediction

## Abstract

A widely used method for prediction of complex traits in animal and plant breeding is “genomic best linear unbiased prediction” (GBLUP). In a quantitative genetics setting, BLUP is a linear regression of phenotypes on a pedigree or on a genomic relationship matrix, depending on the type of input information available. Normality of the distributions of random effects and of model residuals is not required for BLUP but a Gaussian assumption is made implicitly. A potential downside is that Gaussian linear regressions are sensitive to outliers, genetic or environmental in origin. We present simple (relative to a fully Bayesian analysis) to implement robust alternatives to BLUP using a linear model with residual *t* or Laplace distributions instead of a Gaussian one, and evaluate the methods with milk yield records on Italian Brown Swiss cattle, grain yield data in inbred wheat lines, and using three traits measured on accessions of *Arabidopsis thaliana*. The methods do not use Markov chain Monte Carlo sampling and model hyper-parameters, viewed here as regularization “knobs,” are tuned via some cross-validation. Uncertainty of predictions are evaluated by employing bootstrapping or by random reconstruction of training and testing sets. It was found (e.g., test-day milk yield in cows, flowering time and FRIGIDA expression in *Arabidopsis*) that the best predictions were often those obtained with the robust methods. The results obtained are encouraging and stimulate further investigation and generalization.

## 1. Introduction

Arguably, the most widely used method for genome-enabled prediction of complex traits in agriculture is “genomic best linear unbiased prediction,” better known by its acronym GBLUP (Van Raden, 2008). The method adapts a standard mixed effects linear model for obtaining pedigree-based best linear unbiased predictions (ABLUP, hereinafter) of unknown genotypic or breeding values of plants or animals, to a situation where each in a set of individuals possesses multiple-marker DNA information. The molecular markers, typically single nucleotide polymorphisms (SNP), are used as covariates in the linear statistical model, e.g., the first application of GBLUP to sequence data was by Ober et al. (2012).

BLUP (Goldberger, 1962) has been evaluated extensively, including its properties, computational techniques and relationships with other methods (Henderson, 1973; Searle, 1974; Harville, 1976; Robinson, 1991). Prior to the advent of DNA information, genetic evaluation of animals and plants (e.g., Bernardo, 2002) had been carried out via BLUP constructed using a covariance matrix among additive genotypic values of individuals that was proportional to **A**, known as numerator relationship matrix and derived from a pedigree (Henderson, 1976). The innovation in GBLUP is that **A** is replaced by **G**, a marker-based matrix of genomic pair-wise similarities known as “genomic relationship matrix,” of which there are many variants (Van Raden, 2008; Astle and Balding, 2009; Legarra, 2016; Wang et al., 2017); further, the additive genetic variance $\sigma_{a}^{2}$ in ABLUP is replaced by $\sigma_{g}^{2},$ the “genomic variance” (Yang et al., 2010; de los Campos et al., 2015; Lehermeier et al., 2017; Speed et al., 2017). There are two potential advantages of GBLUP over ABLUP. First, the expected similarities in ABLUP calculated on the basis of some idealized evolutionary process (e.g., random mating, absence of selection) are replaced by realized similarities in GBLUP constructed under weaker assumptions. Second, the realized similarities can be pair-specific in GBLUP as opposed to family-specific in ABLUP. In the latter, for example, the expected additive relationship between members of any pair of full-sibs is $\frac{1}{2},$ whereas in GBLUP similarity may vary over pairs of full-sibs.

BLUP can be used in cross-sectional, longitudinal and multiple-trait settings. The flexibility of the method, coupled with concomitant development of a computational machinery applicable to very large data sets, made BLUP widely adopted in animal breeding (Gianola and Rosa, 2015; Weigel et al., 2017). ABLUP and GBLUP can be interpreted as linear regressions of phenotypes on a pedigree or on a genomic relationship matrix, respectively (de los Campos et al., 2009). While normality of the distributions of random effects and of model residuals is not required for BLUP, a Gaussian assumption is made implicitly. The predictor requires information on up to second moments only and all marginal and conditional distributions induced by the model can be derived using normal distribution theory. Further, variance and covariance components needed for BLUP are often estimated using maximum likelihood or Bayesian methods employing Gaussian assumptions, making the dependency of implementations on the multivariate normal distribution patent. However, neither ABLUP nor GBLUP can be considered as robust regression methods. A potential downside is that linear regression methods under Gaussian assumptions are sensitive to outliers (Hampel et al., 1986; Lange et al., 1989; Seber and Lee, 2003), in our case genetic or environmental in origin.

Much research in statistical science has been devoted to developing “robust” regression methods, e.g., Rousseeuw and Leroy (2003). Sensitivity of inference with respect to outlying data points was also recognized in quantitative genetics, and some studies evaluated application of thick-tailed distributions in mixed linear models, especially the *t* distribution and its asymmetric versions (Strandén, 1996; Strandén and Gianola, 1998, 1999; Rosa et al., 2003, 2004; Cardoso et al., 2005; Kizilkaya and Tempelman, 2005; Varona et al., 2006). In animal breeding outliers are often due to performance records of animals receiving undeclared (non-random) preferential treatment: individuals perceived as “better” receive better management. Outlying observations also arise due to concealed sub-structure or underlying heterogeneity, e.g., fertility gradients in plant fields not accounted for in experimental design or spatial model employed for data analysis. In such instances, Gaussian models may not provide an accurate representation of data generating mechanisms.

One property of the *t* distribution or of any of the members of the family of scaled normal distributions applied to residuals (Andrews and Mallows, 1974) is the ability to downweigh automatically observations perceived (by the model) as outliers. Instead of removing outliers prior to analysis via *ad*−*hoc* rules, which results in a loss of information, all observations enter into a robust statistical procedure. An issue associated with removal of outliers is that the uncertainty associated with the exclusion process is not taken into account (Lange et al., 1989).

Another statistical distribution that has been suggested for robust regression analysis is the double exponential or Laplace (Forsythe, 1972; Draper and Smith, 1998). Forsythe (1972) searched for a solution that would produce a least-squares regression mean squared error not much larger than what is obtained when normality holds, but smaller when it does not. The Laplace distribution has been used as prior for regression coefficients in a method called the Bayesian LASSO (Park and Casella, 2008). However, neither the Bayesian LASSO nor the “original LASSO” (Tibshirani, 1996) protect from outliers because normality of the residual distribution is assumed implicitly or explicitly.

A thick tailed residual distribution may produce results that differ importantly from those obtained from an analysis conducted with normally distributed errors. For example, Cardoso et al. (2005) analyzed over 20,000 postweaning body weight gains in cattle using Bayesian linear models with Gaussian or *t*−distributed residuals and compared the respective pedigree-based predictions. Rank correlations between posterior means of additive genetic effects obtained with the two residual distributions ranged from 0.91 to 0.93 over breed groups, but were much lower (0.29–0.57) when focus was on the top 10% of the animals. Their results provide evidence of usefulness of thick-tailed residual distributions in animal breeding.

The most flexible way of fitting a *t* or Laplace residual distribution in a linear or nonlinear model is via a Bayesian Markov chain Monte Carlo (MCMC) analysis. After priors are elicited, the three parameters of the *t* distribution (mean, typically set to 0, scale and degrees of freedom ν) may be inferred from posterior samples. However, the sampling process (especially that for ν) is tricky and computationally intensive and does not lend itself to routine genetic evaluation in animal and plant breeding industries, a large scale computing exercise that needs to be carried out recurrently and fast. Any Bayesian MCMC scheme requires evaluation of effects of hyper-parameters on inference, identification of suitable proposal distributions when conditional posteriors are not in standard form and careful assessment of Monte Carlo error, as it it may happen that sampling error overwhelms statistical signals from the data. Bayesian MCMC methods applied to highly dimensional posterior distributions require meticulous convergence analysis, which is often lacking in practice.

The objective of this paper is to present relatively simple to implement robust alternatives to BLUP using a linear model with residual *t* or Laplace distributions instead of a Gaussian one, and to evaluate the methods using three distinct data sets, to provide proof-of-concept. The approach is MAP, standing for “maximum *a posteriori* prediction”; TMAP and LMAP are the respective acronyms when the *t* or the Laplace distributions are employed. In section 2 the Gaussian mixed effects linear model is reviewed to establish notation, followed by a description of TMAP and LMAP that includes iterative algorithms for calculating point predictors. Section 3 suggests approaches for controlling shrinkage that are “best” in predictive settings. DATA provides a brief description of the information employed in the analysis: milk yield records on Italian Brown Swiss cattle, grain yield in inbred wheat lines, and three traits measured on accessions of *Arabidopsis* *thaliana*. Section 5 presents analyses of model fitting aspects and of predictive power of the various methods entertained. A discussion and concluding comments are presented in the section 6. Algebraic details are in Appendices given at the end of the manuscript and some additional information is available in Supplementary Files.

## 2. Models and assumptions

### 2.1. Linear model with gaussian residuals

A standard univariate mixed effects linear model for quantitative genetic analysis is

(1) y=Wα+Zg+e,

where **y** is an *n* × 1 vector of phenotypic measurements; **α** is an *f*×1 vector of fixed regression coefficients and **W** is an *n*×*f* known incidence matrix with rank *f*; **g** is an *r*×1 randomly distributed vector of genetic (typically additive) effects and **Z** is an *n*×*r* known incidence matrix, and **e** is a vector of residuals. Often, it is assumed that

(2) $$\left(\begin{array}{c} g\\ e \end{array}\right)\sim N\left(\left[\begin{array}{c} 0\\ 0 \end{array}\right],\left[\begin{array}{cc} K\sigma_{g}^{2} & 0\\ 0 & I\sigma_{e}^{2} \end{array}\right]\right),$$

where **K** is a positive semi-definite symmetric similarity matrix (**A** or **G** in ABLUP and GBLUP, respectively) and $\sigma_{g}^{2}$ is a genetic or genomic variance component; **I** is an *n*×*n* identity matrix and $\sigma_{e}^{2}$ is the variance of the residual distribution. Let $h^{2}=\frac{\sigma_{g}^{2}}{\sigma_{g}^{2}+\sigma_{e}^{2}}$ be the coefficient of heritability (“genomic” heritability, $h_{g}^{2}$, when **K** is built using molecular markers); let $\lambda =\frac{\sigma_{e}^{2}}{\sigma_{g}^{2}}=\frac{1-h_{g}^{2}}{h_{g}^{2}}$, and $V=ZKZ^{\prime }\sigma_{g}^{2}+I\sigma_{e}^{2}$ be the *n*×*n* phenotypic variance-covariance matrix.

Under this model, *BLUP*(**g**) is given by

(3) $$\hat{g}=Cov(g,y^{\prime })V^{-1}\left(y-W\tilde{\alpha }\right)=\sigma_{g}^{2}KZ^{\prime }V^{-1}\left(y-W\tilde{\alpha }\right),$$

where $\hat{a}={\left(W^{\prime }V^{-1}W\right)}^{-1}\left(W^{\prime }V^{-1}y\right)$ is the best linear unbiased estimator (*BLUE*) of the fixed effects and also their maximum likelihood estimator under the normality assumption (Equation 2). *BLUE*(**α**) and *BLUP*(**g**) can also be computed simultaneously by solving the mixed model equations (Henderson, 1975)

(4) $$\left[\begin{array}{cc} W^{\prime }W & W^{\prime }Z\\ Z^{\prime }W & Z^{\prime }Z+K^{-1}\lambda \end{array}\right]\left[\begin{array}{c} \hat{\alpha }\\ \hat{g} \end{array}\right]=\left[\begin{array}{c} W^{\prime }y\\ Z^{\prime }y \end{array}\right].$$

If there are no fixed effects in the model and **Z** = **I**, then $\hat{g}={\left(I+K^{-1}\lambda \right)}^{-1}y.$ It is assumed hereinafter that **K**^−1^ exists; BLUP exists even when **K** is singular, provided that **V** has a unique inverse.

### 2.2. Linear model with univariate-*t* residuals

#### 2.2.1. Setting

Assume that all components of **e** are mutually independent but not necessarily identically distributed as $e_{i}\sim t_{\nu }\left(0,\frac{\tau_{e}^{2}}{n_{i}},\nu \right);$ *i* = 1, 2, …, *n*. Here, 0 is the mean of the *t*−distribution; $\frac{\tau_{e}^{2}}{n_{i}}$ is a scale parameter specific to observation *i* such that $Var\left(e_{i}\right)=\frac{\nu }{\nu -2}\frac{\tau_{e}^{2}}{n_{i}}=\sigma_{e_{i}}^{2};$ *n*_*i*_ is a measure of intensiveness of recording on an individual or line (e.g., number of clones) or of degree of replication (e.g., if *y*_*i*_ is some average *n*_*i*_ could be the number of plots in which a line has been planted, or the number of daughters with milk records of a dairy bull); ν>0 is a possibly unknown positive “degrees of freedom” parameter. When ν → ∞,the *t* distribution converges to a normal one and $\sigma_{e_{i}}^{2}\to \frac{\tau_{e}^{2}}{n_{i}}$ (Lange et al., 1989). Since $y_{i}=w_{i}^{\prime }\alpha +z_{i}^{\prime }g+e_{i}$ is a linear combination of *e*_*i*_,conditionally on **g** one has that $y_{i}\sim t_{\nu }\left(\mu_{i},\frac{\tau_{e}^{2}}{n_{i}},\nu \right)$ where $\mu_{i}=w_{i}^{\prime }\alpha +z_{i}^{\prime }g$ (Zellner, 1971; Box and Tiao, 1973); $w_{i}^{\prime }$ and $z_{i}^{\prime }$ , are the *i*th rows of **W** and **Z**,respectively. Note that $h_{g}^{2}=\frac{\sigma_{g}^{2}}{\sigma_{g}^{2}+\frac{\nu }{\nu -2}\tau_{e}^{2}}.$

Keep the Gaussian assumption made in Equation (2) only for the distribution of **g** and continue assuming that **g** and **e** are independent. The joint density of **y** and **g** is now

(5) $$\begin{array}{l} p\left(y,g|\alpha ,\tau_{e}^{2},\sigma_{g}^{2},\nu \right)=\prod_{i\;=\;1}^{n}\frac{\Gamma \left[\frac{\left(\nu +1\right)}{2}\right]}{\;}\\ \sqrt{\nu \frac{\tau_{e}^{2}}{n_{i}}\pi }\Gamma \left(\frac{\nu }{2}\right){\left[1+\frac{n_{i}}{\tau_{e}^{2}\nu }{\left(y_{i}-\mu_{i}\right)}^{2}\right]}^{-\frac{\left(\nu +1\right)}{2}}\\ \times {\left|2\pi K\sigma_{g}^{2}\right|}^{-\frac{1}{2}}\exp\left(-\frac{1}{2\sigma_{g}^{2}}g^{\prime }K^{-1}g\right). \end{array}$$

If $\tau_{e}^{2}$, $\sigma_{g}^{2}$ and ν are taken as known (i.e., given some values) and a flat prior is adopted for **α**, (5) leads to the conditional posterior density (Gianola and Fernando, 1986; Sorensen and Gianola, 2002)

(6) $$\begin{array}{l} p\left(g,\alpha |\tau_{e}^{2},\sigma_{g}^{2},\nu ,y\right)\propto \prod_{i\;=\;1}^{n}{\left[1+\frac{n_{i}}{\tau_{e}^{2}\nu }{\left(y_{i}-\mu_{i}\right)}^{2}\right]}^{-\frac{\left(\nu +1\right)}{2}}\\ \exp\left(-\frac{1}{2\sigma_{g}^{2}}g^{\prime }K^{-1}g\right). \end{array}$$

#### 2.2.2. Maximum a posteriori point estimation (TMAP)

It is shown in Appendix A that the **α** and **g** components of the joint mode of the posterior distribution with density function (6) can be found using the iteration

(7) $$\left[\begin{array}{c} \alpha^{\left[t+1\right]}\\ g^{\left[t+1\right]} \end{array}\right]={\left[\begin{array}{cc} W^{\prime }D^{\left[t\right]}W & W^{\prime }D^{\left[t\right]}Z\\ Z^{\prime }D^{\left[t\right]}Z & Z^{\prime }D^{\left[t\right]}Z+\frac{\lambda^{\prime }\nu }{\left(\nu +1\right)}K^{-1} \end{array}\right]}^{-1}\left[\begin{array}{c} W^{\prime }D^{\left[t\right]}y\\ Z^{\prime }D^{\left[t\right]}y \end{array}\right],$$

where *t* denotes round of iteration, $\lambda^{\prime }=\frac{\tau_{e}^{2}}{\sigma_{g}^{2}}=\lambda \frac{\nu -2}{\nu }$, and **D**^[*t*]^ is an *n*×*n* diagonal matrix with typical element

(8) $$d_{i}=\frac{n_{i}}{\left[1+\frac{{\left(y_{i}-\mu_{i}^{\left[t\right]}\right)}^{2}}{\frac{\tau_{e}^{2}}{n_{i}}\nu }\right]};i\;=\;1,2,...,n.$$

Observe that *d*_*i*_ decreases as *y*_*i*_ departs further from its conditional (given **g**) expectation $\mu_{i}=w_{i}^{\prime }\alpha +z_{i}^{\prime }g$ and as ν decreases. Also, as ν goes to infinity (the *t* distribution approaching normality) *d*_*i*_ moves toward *n*_*i*_,which is the weight assigned to an observation in a Gaussian regression model. After λ, ν and $\tau_{e}^{2}$ are elicited in some manner (see the section 3), starting values for the iteration (*t* = 0) for **α** and **g** could be, e.g., those found with a mixed effects model under Gaussian assumptions. The representation of the algorithm is as in Thompson (1979) Gianola (1982), and Gianola and Foulley (1983).

Appendix A presents the more involved Newton-Raphson (NR) iteration. NR requires more calculations per iterate but typically converges faster to a stationary point than functional iteration (Equation 7). Also, NR provides a basis for constructing approximate posterior credibility intervals for linear combinations of **α** and **g** using a standard Gaussian approximation to the conditional posterior distribution with density (Equation 6): the inverse of the negative Hessian matrix gives the approximate variance-covariance matrix of the unknowns. After solutions converge (denoted as *t* = ∞)

(9) $$\hat{Var}\left(\left[\begin{array}{c} \alpha \\ g \end{array}\right]|\tau_{e}^{2},\sigma_{g}^{2},\nu ,y\right)\approx {\left(-{\left[\begin{array}{c} \frac{\partial^{2}L\left(g,\alpha \right)}{\partial \alpha \partial \alpha^{\prime }}\;\frac{\partial^{2}L\left(g,\alpha \right)}{\partial \alpha \partial g^{\prime }}\\ \frac{\partial^{2}L\left(g,\alpha \right)}{\partial g\partial \alpha^{\prime }}\;\frac{\partial^{2}L\left(g,\alpha \right)}{\partial g\partial g^{\prime }} \end{array}\right]}_{\begin{array}{c} \alpha =\alpha^{\left[\infty \right]}\\ g=g^{\left[\infty \right]} \end{array}}\right)}^{-1}\text{​​},$$

where *L*(**g**, **α**) is the logarithm of Equation (6). For the *t*−model considered here

(10) $$\begin{array}{l} \hat{Var}\left(\left[\begin{array}{c} \alpha \\ g \end{array}\right]|\tau_{e}^{2},\sigma_{g}^{2},\nu ,y\right)\\ \approx \tau_{e}^{2}\frac{\nu }{\left(\nu +1\right)}{\left[\begin{array}{cc} W^{\prime }Q^{\left[\infty \right]}W & W^{\prime }Q^{\left[\infty \right]}Z\\ Z^{\prime }Q^{\left[\infty \right]}W & Zy^{\prime }Q^{\left[\infty \right]}Z+\lambda^{\prime }y\frac{\nu }{\left(\nu +1\right)}K^{-1} \end{array}\right]}^{-1}, \end{array}$$

where **Q** = **D** − **2DSD**, and **S** = *Diag {si}* is an *n* × *n* diagonal matrix with $s_{i}=\frac{{\left(y_{i}-\mu_{i}\right)}^{2}}{\tau_{e}^{2}\nu }$. As ν → ∞, **Q** → **D** and **D** → *Diag {ni}*. In the limit,

(11) $$Var\left(\left[\begin{array}{c} \alpha \\ g \end{array}\right]|\tau_{e}^{2},\sigma_{g}^{2},y\right)=\tau_{e}^{2}{\left[\begin{array}{cc} W^{\prime }NW & WNZ\\ ZNW & Z^{\prime }NZ+\lambda^{\prime }K^{-1} \end{array}\right]}^{-1},$$

gives the exact variance-covariance matrix of the conditional posterior distribution for a model with Gaussian residuals; **N** = *Diag*{*n*_*i*_}; at ν = ∞ then $\tau_{e}^{2}=\sigma_{e}^{2}.$

#### 2.2.3. Special case: zero-means model

Phenotypes are often pre-corrected for effects of systematic sources of variation, such as age and sex of the individual, or year-season of measurement, and then centered so that the sample mean is 0. There would not be any fixed effects so the model becomes **y** = **Zg**+**e**. Further, it is not uncommon in genome-enabled prediction to encounter data sets where all *n* cases have been genotyped, so that **Z** = **I** and therefore **y** = **g**+**e**. In this situation iteration (7) takes the form

(12) $$g^{\left[t+1\right]}={\left[D^{\left[t\right]}+\frac{\lambda^{\prime }\nu }{\left(\nu +1\right)}K^{-1}\right]}^{-1}D^{\left[t\right]}y={\left[I+\frac{\lambda^{\prime }\nu }{\left(\nu +1\right)}D^{\left[t\right]-1}K^{-1}\right]}^{-1}\text{​}y,$$

where **D**^[*t*]^ now has typical element

(13) $$d_{i}=\frac{n_{i}}{\left[1+\frac{{\left(y_{i}-g_{i}^{\left[t\right]}\right)}^{2}}{\frac{\tau_{e}^{2}}{n_{i}}\nu }\right]}.$$

If *n*_*i*_ = 1,the “weights” *d*_*i*_ are at most equal to 1. If ν goes to infinity *d*_*i*_→*n*_*i*_ and the solution is explicit: $\hat{g}={\left(I+\lambda D^{-1}K^{-1}\right)}^{-1}Dy.$ Further, if *n*_*i*_ = 1∨*i*,the expression becomes the standard representation of GBLUP $\hat{g}={\left(I+\lambda K^{-1}\right)}^{-1}y$.

For finite ν,there are three factors in Equation (12) that control shrinkage of solutions and attenuation of data points: the variance ratio λ′, the degrees of freedom υ and the scale parameter $\tau_{e}^{2}$: shrinkage increases with λ′ whereas attenuation of phenotypes is stronger when υ and $\tau_{e}^{2}$ are small. The impact of observation *i* depends on the discrepancy between observed *y*_*i*_ and fitted ($g_{i}^{\left[t\right]}$) genotypes; if the fitted residual is large, case *i* receives less weight in the analysis than otherwise. The representation to the right of Equation (12) Illustrates how regularization and attenuation work together: fitted genotypic values associated with cases receiving low weights are more strongly shrunk to 0 (the prior mean of *g*_*i*_) than cases with larger weights. This feature of TMAP confers robustness relative to GBLUP.

In the zero-means model, the approximation to the variance-covariance matrix of the conditional posterior distribution of **g** (Appendix A) is

(14) $$\hat{Var}\left(g|\tau_{e}^{2},\sigma_{g}^{2},\nu ,y\right)\approx \tau_{e}^{2}\frac{\nu }{\left(\nu +1\right)}{\left[Q^{\left[\infty \right]}+\lambda^{\prime }\frac{\nu }{\left(\nu +1\right)}K^{-1}\right]}^{-1},$$

where $Q=Diag\left\{d_{i}-2d_{i}^{2}s_{i}\right\}$ and $s_{i}=\frac{{\left(y_{i}-g_{i}\right)}^{2}}{\tau_{e}^{2}\nu }.$

### 2.3. Linear model with laplace residuals

#### 2.3.1. Setting

Assume now that observations are (conditionally) independently distributed as $y_{i}|\mu_{i},\sigma_{e}^{2}\sim Laplace(\mu_{i},\frac{\sigma_{e}^{2}}{n_{i}})$. Often, the density of the Laplace or double exponential (DE) distribution is written as

(15) $$p\left(y_{i}|\mu_{i},\delta \right)=\frac{\sqrt{n_{i}}}{2\delta }\exp\left(-\frac{\sqrt{n_{i}}\left|y-\mu_{i}\right|}{\delta }\right);i\;=\;1,2,...,n,$$

where $\delta =\sqrt{\frac{\sigma_{e}^{2}}{2}}$ is a parameter that relates to spread of the distribution. The probability density function for the sampling model is then

(16) $$p\left(y|\alpha ,g,\sigma_{e}^{2}\right)=\prod_{i\;=\;1}^{n}\sqrt{\frac{n_{i}}{2\sigma_{e}^{2}}}\exp\left(-\frac{\sqrt{n_{i}}\left|y_{i}-\mu_{i}\right|}{\sqrt{\frac{\sigma_{e}^{2}}{2}}}\right).$$

Adopting again a flat prior for **α** and the Gaussian prior in (2), the log-conditional posterior density of **g** and **α** (*C* is an additive constant) is

(17) $$\begin{array}{l} L_{DE}=\log\left[p\left(g,\alpha |\sigma_{e}^{2},\sigma_{g}^{2},y\right)\right]\\ \;=C-\frac{1}{\sqrt{\frac{\sigma_{e}^{2}}{2}}}\sum_{i\;=\;1}^{n}\sqrt{n_{i}}\left|y_{i}-\mu_{i}\right|-\frac{1}{2\sigma_{g}^{2}}g^{\prime }K^{-1}g. \end{array}$$

#### 2.3.2. Maximum a posteriori estimation (LMAP)

As shown in Appendix B (see Equation 58 in Appendix), a mode of the conditional posterior density above can be located using an iterative scheme similar to (7) but with a diagonal matrix **M**^[*t*]^ replacing **D**^[*t*]^. **M**^[*t*]^ has diagonal elements

(18) $$m_{i}=\frac{\sqrt{n_{i}}}{\left|y_{i}-\mu_{i}^{\left[t\right]}\right|};i\;=\;1,2,...,n,$$

and with $\omega =\frac{\sqrt{\frac{\sigma_{e}^{2}}{2}}}{2\sigma_{g}^{2}}=\frac{\delta }{2\sigma_{g}^{2}}$ as a regularization parameter replacing $\frac{\lambda^{\prime }\nu }{\left(\nu +1\right)}$ in Equation (7).

If $\sigma_{g}^{2}$ is a “genomic” variance (de los Campos et al., 2015), $h_{g}^{2}$ is genomic heritability and $\sigma_{y}^{2}$ is the phenotypic variance

(19) $$\omega =\frac{\sqrt{\frac{\left(1-h_{g}^{2}\right)\sigma_{y}^{2}}{2}}}{2h_{g}^{2}\sigma_{y}^{2}}.$$

The value of ω can be approximated directly from estimates of $\sigma_{e}^{2}$ and $\sigma_{g}^{2},$ or from knowledge of trait “genomic heritability” and of the phenotypic variance. If phenotypes are scaled to have unit variance, one may set $\sigma_{y}^{2}=1$ in the expression above.

The variance-covariance matrix of the conditional posterior distribution can be approximated (Appendix B) as

(20) $$\hat{Var}\left(\left[\begin{array}{c} \alpha \\ g \end{array}\right]|\sigma_{e}^{2},\sigma_{g}^{2},y\right)=\frac{\sqrt{\frac{\sigma_{e}^{2}}{2}}}{2}{\left[\begin{array}{cc} W^{\prime }M^{\left[\infty \right]}W & W^{\prime }M^{\left[\infty \right]}Z\\ Z^{\prime }M^{\left[\infty \right]}W & Z^{\prime }M^{\left[\infty \right]}Z+\omega K^{-1} \end{array}\right]}^{-1}.$$

#### 2.3.3. Special case: zero-means model

Recall that **Z** = **I** and **y** = **g**+**e**. The iteration becomes

(21) $$g^{\left[t+1\right]}={\left[M^{\left[t\right]}+\omega K^{-1}\right]}^{-1}M^{\left[t\right]}y={\left[I+\omega {\left(M^{\left[t\right]}\right)}^{-1}K^{-1}\right]}^{-1}y$$

so phenotype *i* “effectively” enters into the analysis as $m_{i}^{\left[t\right]}y_{i}=\sqrt{n_{i}}\frac{y_{i}}{\left|y_{i}-g_{i}^{\left[t\right]}\right|}.$ Case-specific regularization is effected via $\omega \frac{\left|y_{i}-g_{i}\right|}{\sqrt{n_{i}}},i\;=\;1,2,...,n;$ genotypic values of individuals with phenotypic values departing markedly from their conditional expectation (*g*_*i*_) are more heavily shrunk toward zero.

## 3. Predictive assessment of regularization parameters

### 3.1. Difficulties with bayesian and likelihood-based methods

Recall that our objective is to develop procedures suitable for routine application in a reasonably practical manner. A fully Bayesian approach (under standard priors) to inferring $\alpha ,g,\;\sigma_{g}^{2},\tau_{e}^{2}$ and ν is straightforward in TMAP because the fully-conditional distributions needed for implementing a Gibbs sampler are recognizable, save for that of the degrees of freedom (Strandén and Gianola, 1999; Sorensen and Gianola, 2002; Rosa et al., 2003). The MCMC algorithm in a model with a DE residual distribution is not easy to run either.

An alternative to Bayesian MCMC is maximum likelihood, although it presents difficulties as well. For instance, consider estimating $\theta ={\left(\alpha ,\;\tau_{e}^{2},\sigma_{g}^{2},\nu \right)}^{\prime }$ in the *t*-model by maximum likelihood via the EM algorithm (Dempster et al., 1977). It is well known (Box and Tiao, 1973; Andrews and Mallows, 1974; Lange et al., 1989) that a *t* distribution can be generated by mixing a normal distribution with a randomly varying variance ($\sigma_{e_{i}}^{2},$ say), over inverted Gamma or scaled-inverted chi-square distributions, depending on the parameterization adopted. Lange et al. (1989) described the EM algorithm for a fixed linear regression model with *t*−distributed residuals, i.e., with both **α** and **g** as fixed parameters, and with the $\sigma_{e_{i}}^{2}$ variables treated as “missing” data. In such model, the only “problematic” parameter is ν. In our setting, the missing data include not only the auxiliary variances $\sigma_{e_{i}}^{2}$ used for creating a *t* distribution but the **g** vector of genetic effects as well. The E-step of the algorithm encounters a difficulty when **g** is random (even with ν fixed at some value): the conditional distribution of **g** given **y** (at the current value of the parameters) cannot be written in closed form, so estimation requires embedding a Monte Carlo step, negating the simplicity of EM.

The model with a Laplace residual distribution is not very tractable either. Perhaps this explains why the DE distribution has not appeared in the literature of non-Bayesian random effects models more often. In short, neither maximum likelihood nor Bayesian MCMC are appealing for our purposes. Approaches that are simpler from a computational perspective and targeted to prediction tasks are presented later in the paper.

### 3.2. Evaluation via cross-validation

Assume hereinafter that markers are used for constructing **K**. In GBLUP, $\lambda =\frac{1-h_{g}^{2}}{h_{g}^{2}}$ is the counterpart of ω in Equation (19) for LMAP and of $\lambda^{\prime \prime }=\lambda^{\prime }\frac{\nu }{\nu +1}$ in TMAP, as shown in Equation (7). The relationship between heritability and regularization parameters λ,λ^′′^ and ω (assuming the phenotypic variance is 1 in the latter) is shown in Figure S1. At low heritability (top panel), the DE distribution of LMAP exerts less regularization (lower ω than λ or λ^′′^) than the normal or *t*−distributions (ν = 4 in Figure S1). At intermediate heritability (middle panel), regularization is also less strong for LMAP and TMAP than for GBLUP, and the methods continue approaching each other as as heritability increases. For heritability larger than 0.60, LMAP induces less shrinkage than GBLUP or TMAP until $h_{g}^{2}\approx 0.80,$ point at which it it intersects with TMAP, and later with GBLUP. TMAP shrinks solutions less strongly than GBLUP throughout. Recall, however, that the thick-tailed models also produce datum-specific attenuation, so Figure S1 does not tell the whole story. In LMAP, the impact of a datum is proportional to ${\left|y_{i}-\mu_{i}\right|}^{-1},$ so observations far from fitted values are less influential; in TMAP, attenuation is via *d*_*i*_ whose value is affected by $\left|y_{i}-\mu_{i}\right|$ and also by ν and $\tau_{e}^{2}$. Attenuation has an important effect on predictive outcomes, as illustrated later.

After some validation or cross-validation (CV) scheme is chosen, e.g., a training-testing layout, λ in GBLUP can be varied over a grid of plausible $h_{g}^{2}$ values, using some focal point from the literature or from estimates obtained from the training data (see below); in TMAP and LMAP estimates of $\sigma_{e}^{2}$ and $\sigma_{g}^{2}$ are needed for forming λ′ and ω. The grid search approach informs about the sensitivity of predictions when the dispersion structure changes. The resulting information can be especially valuable for the small (relative to animal breeding) populations used in plant breeding, or for experimental material.

It can be seen in Equations (7) and (8) or in Equations (12) and (13) that values of λ′,ν and of the scale parameter $\tau_{e}^{2}$ (appearing “inside” of *d*_*i*_) are needed in TMAP. The degrees of freedom ν could be varied between, say, 3 (to ensure a finite variance) and 20; values larger than 20 will probably produce predictions similar to those of GBLUP. The scale $\tau_{e}^{2}$ can be assessed using a simple method. Suppose twenty and fifteen values are posed for each of the λ ($h_{g}^{2})$ and ν parameters, respectively. For each of the 300 resulting combinations, training and testing sets can be constructed at random (if feasible) 200 times, say. The residual variance $\sigma_{e}^{2}$ is estimated at each instance of training; given $\sigma_{e}^{2}$ and ν in the grid, $\tau_{e}^{2}$ can be assessed readily and TMAP fitted accordingly. Mean squared errors of prediction and predictive correlations are then evaluated in testing sets and the uncertainty of the predictive performance can be measured from the 200 replications or by using either bootstrapping or by adapting methods presented by Gianola and Schön (2016) and Xu (2017).

Since $\sigma_{e}^{2}$ and possibly $\sigma_{g}^{2}$ would need to be estimated at each training instance, a simple non-iterative method is required. Rao (1970) proposed MINQUE (minimum norm quadratic unbiased estimation) for estimation of variance components using quadratic forms. MINQUE does not assume normality and can be used for models with any random effects or residual distributions. Actually, iterating MINQUE produces REML provided that convergence is in the parameter space. There is no unique MINQUE estimator because estimates depend on the choice of some arbitrary positive weights (e.g., a value of λ or of $h_{g}^{2}$ in the grid); any of such weights produces unbiased estimates (Searle et al., 2006). Since, MINQUE produces training data estimates of $\sigma_{g}^{2}$ as well, ω in LMAP can be formed directly.

MINQUE also allows for fixed effects but we will consider a zero-means model, for simplicity. Let **y** = **g** + **e**,such that *E*(**y**) = **0** and $Var\left(y\right)=G\sigma_{g}^{2}\;+\;I\sigma_{e}^{2}=V;$ in TMAP $\tau_{e}^{2}=\frac{\nu -2}{\nu }\sigma_{e}^{2}$. Make a “guess” about $h_{g}^{2}$ (or λ) and set

(22) $$V_{\text{guess}}=G\sigma_{g,\text{guess}}^{2}+I\sigma_{e,\text{guess}}^{2}=\left(G\lambda_{\text{guess}}^{-1}+I\right)\sigma_{e,\text{guess}}^{2},$$

where $\lambda_{\text{guess}}=\frac{1-h_{\text{guess}}^{2}}{h_{\text{guess}}^{2}}$ is the guess for a point in the grid. Actually, $\sigma_{e,\text{guess}}^{2}$ is not needed for computing MINQUE. Letting $V_{\text{guess}}^{*}=\left(G\lambda_{\text{guess}}^{-1}+I\right)$ MINQUE estimates the unknown variances by solving in one shot (i.e., non-iteratively)

(23) $$\begin{array}{l} \left[\begin{array}{cc} tr\left(GV_{\text{guess}}^{*-1}GV_{\text{guess}}^{*-1}\right) & tr\left(GV_{\text{guess}}^{*-1}V_{\text{guess}}^{*-1}\right)\\ tr\left(GV_{\text{guess}}^{*-1}V_{\text{guess}}^{*-1}\right) & tr\left(V_{\text{guess}}^{*-1}V_{\text{guess}}^{*-1}\right) \end{array}\right]\left[\begin{array}{c} {\hat{\sigma }}_{g}^{2}\\ {\hat{\sigma }}_{e}^{2} \end{array}\right]\\ =\left[\begin{array}{c} y^{\prime }V_{\text{guess}}^{*-1}GV_{\text{guess}}^{*-1}y\\ y^{\prime }V_{\text{guess}}^{*-1}V_{\text{guess}}^{*-1}y\%y \end{array}\right]. \end{array}$$

The expected values of the quadratic forms on the right-hand sides of Equation (23) are

(24) $$\begin{array}{l} E\left(y^{\prime }V_{\text{guess}}^{*-1}GV_{\text{guess}}^{*-1}y\right)=tr\left[V_{\text{guess}}^{*-1}GV_{\text{guess}}^{*-1}\left(G\sigma_{g}^{2}+I\sigma_{e}^{2}\right)\right]\\ \;=tr\left(GV_{\text{guess}}^{*-1}GV_{\text{guess}}^{*-1}\right)\sigma_{g}^{2}\\ \;+\;tr\left(GV_{\text{guess}}^{*-1}V_{\text{guess}}^{*-1}\right)\sigma_{e}^{2}\\ \;=c_{11}\sigma_{g}^{2}+c_{12}\sigma_{e}^{2}, \end{array}$$

and

(25) $$\begin{array}{l} E\left(y^{\prime }V_{\text{guess}}^{*-1}V_{\text{guess}}^{*-1}y\right)=tr\left[V_{\text{guess}}^{*-2}\left(G\sigma_{g}^{2}+I\sigma_{e}^{2}\right)\right]\\ \;=tr\left(GV_{\text{guess}}^{*-2}\right)\sigma_{g}^{2}+tr\left(V_{\text{guess}}^{*-2}\right)\sigma_{e}^{2}\\ \;=c_{21}\sigma_{g}^{2}+c_{22}\sigma_{e}^{2}. \end{array}$$

Using the preceding in Equation (23)

(26) $$E\left(\left[\begin{array}{c} {\hat{\sigma }}_{g}^{2}\\ {\hat{\sigma }}_{e}^{2} \end{array}\right]\right)={\left[\begin{array}{cc} c_{11} & c_{12}\\ c_{21} & c_{22} \end{array}\right]}^{-1}\left[\begin{array}{cc} c_{11} & c_{12}\\ c_{21} & c_{22} \end{array}\right]\left[\begin{array}{c} \sigma_{g}^{2}\\ \sigma_{e}^{2} \end{array}\right]=\left[\begin{array}{c} \sigma_{g}^{2}\\ \sigma_{e}^{2} \end{array}\right],$$

which shows the unbiasedness of MINQUE at any λ_guess_. TMAP requires eliciting the scale parameter $\tau_{e}^{2}$ of the *t*−distribution. An unbiased estimator of $\tau_{e}^{2}$ (given the current ν in the grid) is ${\hat{\tau }}_{e}^{2}=\frac{\nu -2}{\nu }{\hat{\sigma }}_{e}^{2}.$ In LMAP, using (19) one can set $\hat{\omega }=\frac{\sqrt{\frac{{\hat{\sigma }}_{e}^{2}}{2}}}{2{\hat{\sigma }}_{g}^{2}}.$

The predictive algorithm for a training-testing layout would flow as follows:

- Construct a grid of λ and ν values with *B* two-dimensional entries.
- Divide data at random into training and testing sets. Repeat *N*_*rep*_ times, e.g., *N*_*rep*_ = 100.
- Estimate $\sigma_{g}^{2}$ and $\sigma_{e}^{2}$ by MINQUE, estimate $\tau_{e}^{2}$ and ω and fit the models in each of the *B*×*N*_*rep*_ training instances.
- Evaluate predictive correlation and mean-squared error (or any other metric) for each testing set.
- Construct prediction error distributions for each of the *B* points in the grid and determine the optimum predictive performance.

### 3.3. Generalized cross-validation

An approach to tuning regularization parameters in ridge regression, also applicable to GBLUP, consists of employing all data with “generalized cross-validation”; see Craven and Wahba (1979) and Golub et al. (1979) for theoretical foundations in connection with ridge regression and Xu (2017) for an application. For a zero-means GBLUP model, let

(27) $$H_{GBLUP}\left(\lambda \right)={\left(I+K^{-1}\lambda \right)}^{-1},$$

and let the prediction error be ϵ(λ) = **y** − ${\hat{g}}_{G}\left(\lambda \right)=\left[I-H_{GBLUP}\left(\lambda \right)\right]y$. The generalized cross-validation criterion is calculated over the grid of λ−values as

(28) $$\begin{array}{l} GCV_{GBLUP}\left(\lambda \right)=\frac{1}{n}\epsilon {\left(\lambda \right)}^{\prime }\epsilon \left(\lambda \right)/{\left\{\frac{1}{n}tr\left[I-H_{GBLUP}\left(\lambda \right)\right]\right\}}^{2}\\ \;=\frac{1}{n}y^{\prime }{\left[I-H_{GBLUP}\left(\lambda \right)\right]}^{2}y/{\left\{1-{\bar{h}}_{GBLUP}\left(\lambda \right)\right\}}^{2}; \end{array}$$

where $\bar{h}\left(\lambda \right)$ is the average of the diagonal elements of **H**_*GBLUP*_(λ). While GCV is well established theoretically for ridge regression, expressions for TMAP and LMAP do not exist. For GBLUP, the GCV criterion can be calculated at each entry in the grid of values of λ, to locate the setting producing the best expected predicting performance in the sense of minimizing (Equation 28).

Thompson (2001) discussed how GCV could be used in a mixed effects linear model, as well as to how quadratics appearing in such GCV also arise in an algorithm for REML under Gaussian assumptions. The implication is that REML could be computed from the entire data set and used in conjunction with GCV. However, the analogy is not complete: ridge regression (and BLUP) are distribution-free procedures whereas REML is not, and the results may differ. Further, conditioning on a single set of estimates of the regularization parameters does not inform about sensitivity of predictions, as noted earlier.

## 4. Data

Three data sets were used to evaluate if TMAP or LMAP could deliver a better predictive ability than GBLUP. One data set (dairy cattle) is representative of observational learning, i.e., data may be non-random and contaminated due to factors not contemplated in the model. The second data set (*Arabidopsis*) is representative of a situation in which experimental conditions are carefully controlled, as is often the case with model organisms. The third data (wheat) reflects agronomic conditions, where experimental tuning is much finer than in animal breeding because of the possibility of organizing plots into fields and of randomization.

All *Arabidopsis* and data necessary for confirming the conclusions of this article can be downloaded from the R Synbreed package (Wimmer et al., 2012). The wheat data are included in the R package BGLR (Pérez and de los Campos, 2014). The Brown Swiss data is proprietary information owned by private breeders and by Associazione Nazionale Allevatori Razza Bruna Italiana (ANARB), Bussolengo, Italy.

### 4.1. Italian brown swiss cattle

The data came from “Cowability–Cowplus” projects at the University of Padova, Italy. Full descriptions are in Dadousis et al. (2016). Briefly, milk samples from 1,264 Italian Brown Swiss cattle from 85 herds were collected at evening milkings. All samples were collected following milk recording protocols of the Breeders Federation of Trento Province.

Cows were genotyped with the Illumina BovineSNP50 v.2 BeadChip (Illumina Inc., San Diego, CA). Markers included in the analysis were such that call rate > 95%, minor allele frequency > 0.5%, and no deviation from Hardy-Weinberg proportions detected (*p* > 0.001, Bonferroni corrected). Our target trait was “single test-day” milk pre-corrected as in Dadousis et al. (2016), i.e., using ordinary least-squares estimates of effects of class of days in milk of the cow (classes of 30 days each), cow parity (1, 2, 3, ≥4) and herd-day effect (85 levels). See Dadousis et al. (2016) for full details. After edits, *n* =991 cows and *p* = 37,568 SNP were retained. **G** was built with function getG from the BGData library (https://github.com/QuantGen?BGData).

### 4.2. *Arabidopsis*

The *Arabidopsis thaliana* data set described by Atwell et al. (2010) was used. These authors noted that the sample of accessions suggested a complex structure in the population. Such complexity was confirmed by a multi-dimensional scaling analysis conducted by Gianola et al. (2016). The data, available in the R Synbreed package (Wimmer et al., 2012), represents 199 accessions genotyped with a custom Affymetrix 250K SNP chip. As in Wimmer et al. (2012), flowering time (*n* = 194), plant diameter (*n* = 180) and FRIGIDA (*n* = 164) gene expression were chosen as target phenotypes; marker genotypes are pre-edited in the package and 215,947 SNP loci were used in the analysis.

### 4.3. Wheat grain yield

The wheat data in package BGLR (Pérez and de los Campos, 2014) was employed. This data set is well characterized and has also been used, e.g., by Crossa et al. (2010), Gianola et al. (2011), Long et al. (2011), Gianola and Schön (2016), and Gianola et al. (2016). The data came from trials conducted by the International Maize and Wheat Improvement Center (CIMMYT), Mexico. There are 599 wheat inbred lines, each genotyped with 1279 DArT (Diversity Array Technology) markers and planted in four environments. Sample size was *n* = 599 and *p* = 1, 279 was the number of markers. The DArT markers are binary (0, 1) and denote presence or absence of an allele at a marker locus in a given line. The data set also includes a pedigree-derived relationship matrix (**A**). Grain yield was our target.

## 5. Application and evaluation of methods

### 5.1. Brown swiss cattle

#### 5.1.1. Goodness of fit and dispersion parameters

A QQ plot shown in Figure S2 suggests some departure of pre-corrected test-day milk yields from normality, so a linear model with Gaussian residuals may not be adequate. Lack of fit may be reflective of undeclared (unrecorded) differential treatment of cows, but other reasons cannot be ruled out.

Maximum likelihood estimates of dispersion parameters were obtained using the model **y** = **g**+**e**, with $g\sim \;N\left(0,G\sigma_{g}^{2}\right)$ and assuming Gaussian residuals. Estimates were 0.074 (genomic variance), 0.926 (residual variance), and 0.074 (genomic heritability); hence $\hat{\lambda }=12.5$ is the maximum likelihood estimate of the regularization parameter in GBLUP. Our estimate of genomic heritability aligns well with knowledge on heritability of single test-day yield in the population. A grid of 19 genomic heritability values ranging from 0.05 to 0.95 ($h_{\text{guess}}^{2})$ with increments of 0.05 was used to obtain MINQUE estimates of genomic and residual variances,and to assess sensitivity of predictions with respect to variation in variance partitioning. Estimates of the $h_{g}^{2}$ parameter ($h_{\text{guess}}^{2}$ between parentheses) ranged between 0.07 (0.05) and 0.47 (0.95); 17 of the 19 MINQUE estimates were lower than 0.25. Estimates of $h_{g}^{2}$ were always lower than the corresponding “guesses” save for $h_{\text{guess}}^{2}=0.05$. As noted, MINQUE produces unbiased estimates of variance components no matter what $h_{\text{guess}}^{2}$ is (provided the model holds), but their sampling variances increase when guesses depart from true (unknown) values. Our results flag to situations in which the data dispute a guess in the grid, e.g., $h_{\text{guess}}^{2}>0.15$. Our algorithm produces stronger regularization than what would be indicated by any given $h_{\text{guess}}^{2},$ save for 0.05.

#### 5.1.2. Regularization (shrinkage)

We fitted zero-mean linear models with $g\sim \;N\left(0,G\sigma_{g}^{2}\right),$ and with **e** consisting of independent and identically distributed terms that followed either normal, Student's-*t* (ν = 4, 8, 12, 16) or Laplace distributions. Models were fitted at all values of the $h_{\text{guess}}^{2}$ grid indicated above, using MINQUE ($h_{\text{guess}}^{2})$ to obtain estimates of the genomic and residual variance components, for regularization purposes. For the *t*−distribution, the scale parameter was estimated as ${\hat{\tau }}_{e}^{2}=\frac{\nu -2}{\nu }{\hat{\sigma }}_{e}^{2}$ where ${\hat{\sigma }}_{e}^{2}$ was the MINQUE estimate of the residual variance for the appropriate grid entry. For the Laplace distribution, the regularization parameter was formed as $\hat{\omega }=\frac{\sqrt{{\hat{\sigma }}_{e}^{2}/2}}{2{\hat{\sigma }}_{g}^{2}},$ with ${\hat{\sigma }}_{e}^{2}$ and ${\hat{\sigma }}_{g}^{2}$ being MINQUE ($h_{\text{guess}}^{2})$ estimates. Hence, there were 19 (Gaussian), 19 × 4 = 76 Student-*t* models, and 19 Laplace models fitted to the entire cow data set.

In TMAP, iteration (Equation 12) converged in 5 or less rounds for the 76 models fitted, as indicated by the average of *d*−values; subsequent analyses used TMAP solutions at iterate 10 as converged. Minimum, median and maximum *d*−values at convergence were, for example: 0.20, 0.92 and 1.00 ($h_{\text{guess}}^{2}=0.05,$ ν = 4); 0.50, 0.98 and 1.00 ($h_{\text{guess}}^{2}=0.05,$ ν = 16); 0.21, 0.97 and 1.00 ($h_{\text{guess}}^{2}=0.95,$ ν = 4),and 0.55, 0.99 and 1.00 ($h_{\text{guess}}^{2}=0.95,$ ν = 16). As anticipated, observations were assigned lower weights at smaller values of ν and of $h_{\text{guess}}^{2}$; in Gaussian models all observations receive a weight equal to 1. In LMAP, the algorithm converged (average of *m*−values monitored) in 15 or less iterations; subsequent analyses used results from iteration 30.

**Figure 1:**
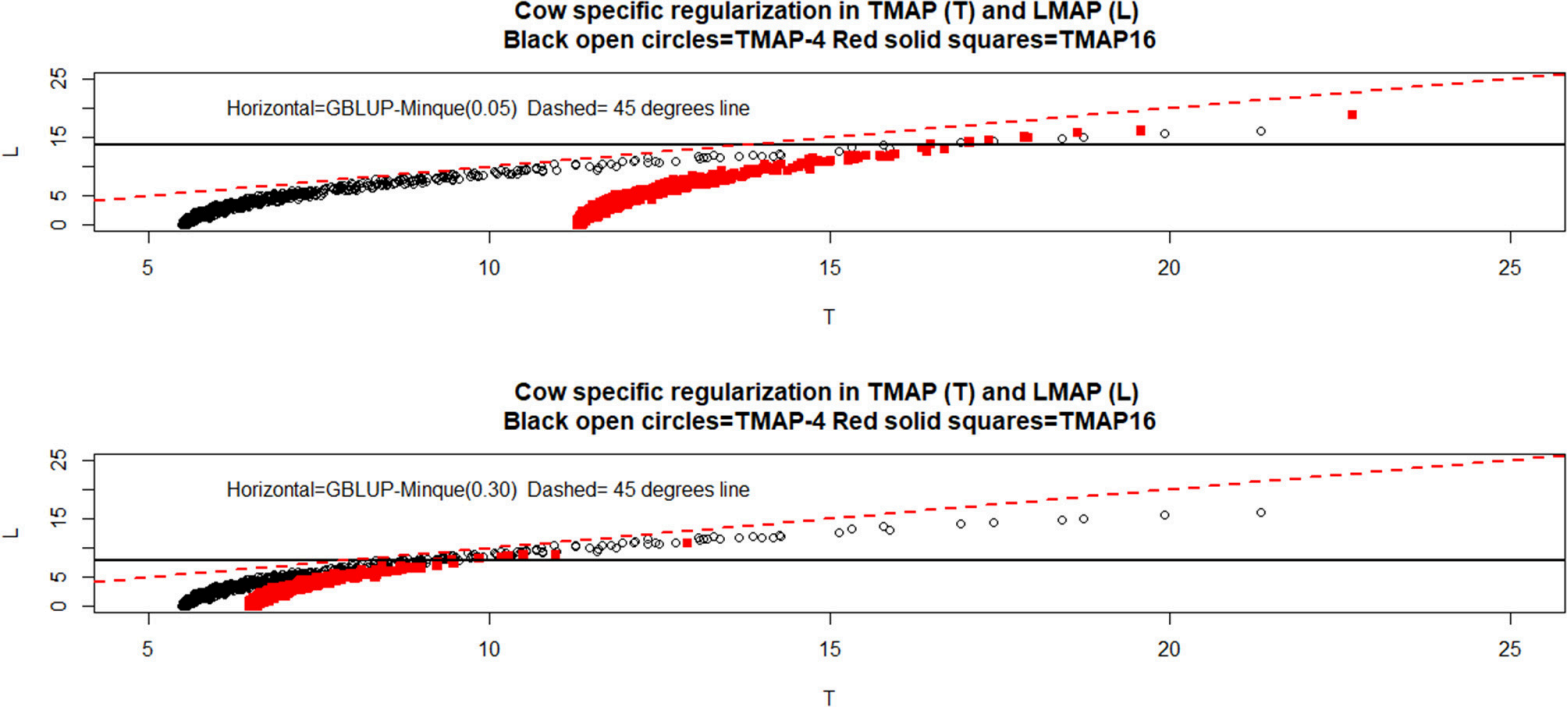
Joint effect of regularization and attenuation (see text) of milk yield test-day records in Italian Brown Swiss cows at two MINQUE “guesses” of genomic heritability. GBLUP: genomic best linear unbiased prediction. TMAP-4 (16): maximum a posteriori with residual *t*—distribution on 4 (16) degrees of freedom. LMAP: maximum a posteriori with a double exponential residual distribution. MINQUE: minimum norm quadratic unbiased estimator of variance components. T and L denote the cow-specific regularization and attenuation in TMAP and LMAP, respectively.

Note from Equations (12) and (21) that case-specific effective shrinkage, i.e., the joint effect of regularization and attenuation, can be measured and compared using the diagonal elements of $\frac{\lambda^{\prime }\nu }{\left(\nu +1\right)}{\text{D}}^{\left[t\right]-1}$ (TMAP) and of ω**M**^[*t*]−1^(LMAP). The relationship between the effective shrinkage produced by the various models depends on several factors, including the value of ν, $h_{\text{guess}}^{2}$ and the closeness of fit. Figure 1 displays relationships between the diagonal elements mentioned previously in TMAP-4 or TMAP-16 (T in the plot) and LMAP (L) for the cow data using $h_{\text{guess}}^{2}=0.05$ and 0.30. The horizontal lines in the plot give the values of λ used in GBLUP (homogeneous shrinkage) as per the appropriate MINQUE estimates; these were 13.72 ($h_{\text{guess}}^{2}=0.05,{\hat{h}}_{g}^{2}=0.068)$ and 7.81 ($h_{\text{guess}}^{2}=0.30,{\hat{h}}_{g}^{2}=0.114).$ T and L values varied concomitantly, but their association was neither linear nor perfect. L was always larger than T, and the effective shrinkage produced by the two methods was not always stronger than in GBLUP, i.e., when paths were below the horizontal lines. The preceding implies that the joint effects of regularization and attenuation are subtle. Note that *T* values from different specifications of ν were more similar at higher heritability (bottom panel).

#### 5.1.3. Model fit

**Figure 2:**
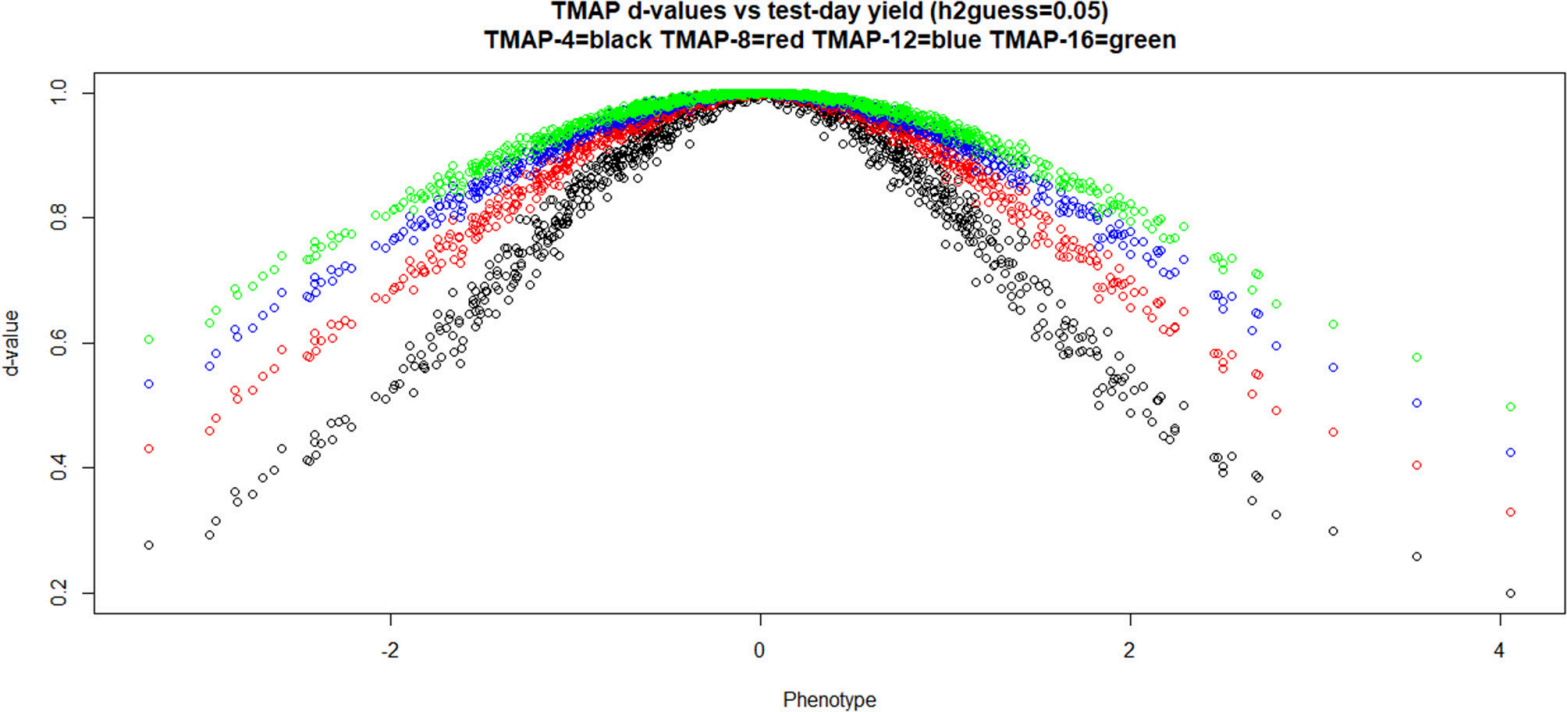
Weights (at the last round of iteration) assigned to individual cow milk yield test-day records in TMAP-4, TMAP-8, TMAP-12, and TMAP-16. MINQUE guess for heritability was 0.05. MINQUE: minimum norm quadratic unbiased estimator of variance components.

Figure 2 illustrates how the TMAP weights assigned to data points varied with phenotypic values (in units of standard deviation, SD) and with the degrees of freedom parameter as observations departed from the mean; as expected, *d*−values increased with ν. Extreme values are more severely discounted than intermediate ones and, remarkably, observations falling within the range ± 1 SD from the mean were also attenuated to varying degrees. Recall that GBLUP assigns a weight equal to 1 to all observations. The *m*−values in LMAP cannot be easily interpreted as weights, so a similar plot was not made for such model.

**Figure 3:**
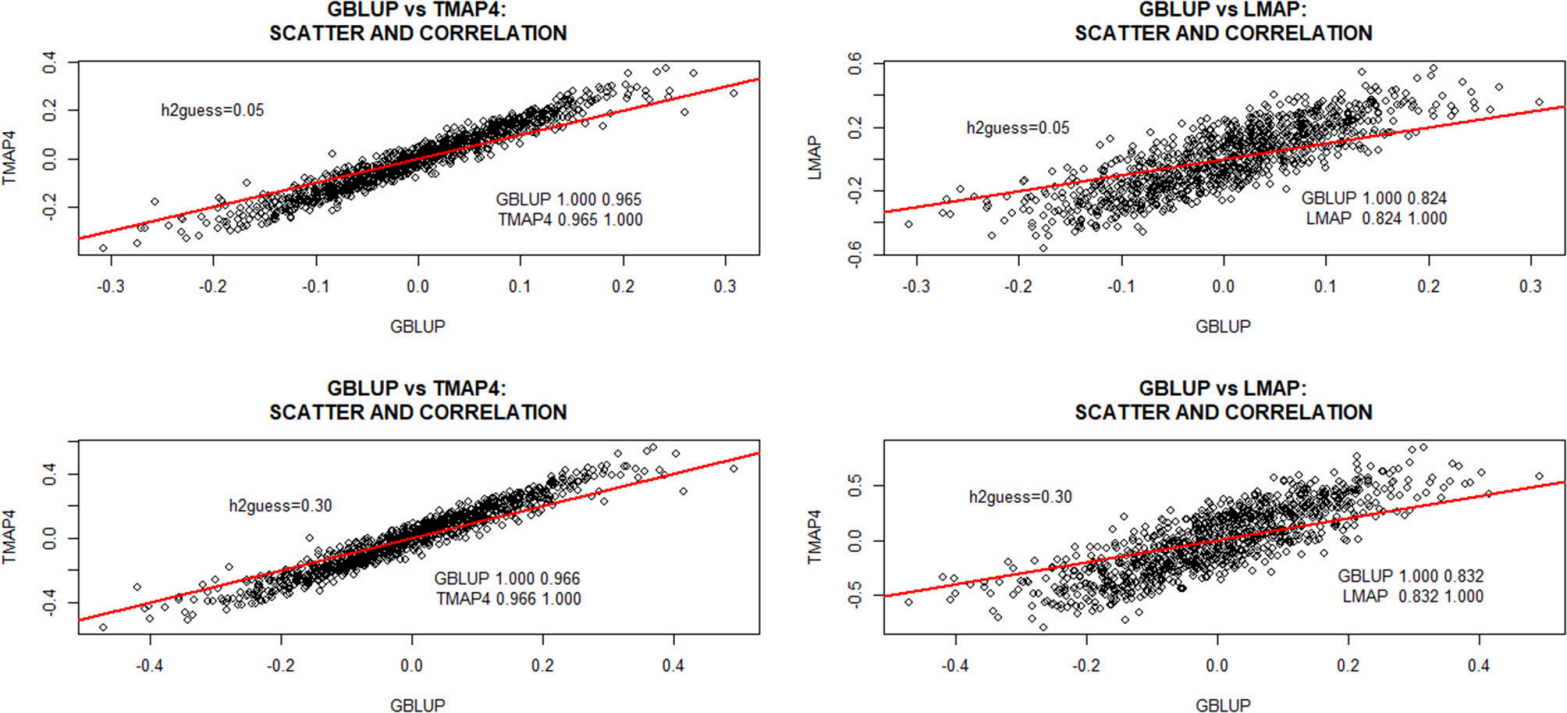
Predicted additive genomic values of milk yield test-day records in Italian Brown Swiss cows and inter-correlations between predictions at two MINQUE “guesses” of genomic heritability. GBLUP: genomic best linear unbiased prediction. TMAP-4: maximum a posteriori with residual *t*—distribution on 4 degrees of freedom. LMAP: maximum a posteriori with a double exponential residual distribution. MINQUE: minimum norm quadratic unbiased estimator of variance components.

For TMAP, the discussion will focus mostly on ν = 4 since differences between TMAP and GBLUP were often more marked at the smallest specification of the degrees of freedom. The scatterplots in Figure 3 depict differences between GBLUP and TMAP-4 or LMAP estimates of additive genomic effects obtained with the entire data set at $h_{\text{guess}}^{2}=0.05$ and 0.30. GBLUP was more strongly correlated with TMAP-4 than with LMAP. Correlations between GBLUP and LMAP were 0.824 and 0.832 for $h_{\text{guess}}^{2}=0.05$ and 0.70, respectively. Correlations (not shown) between TMAP-4 and LMAP were 0.91 ($h_{\text{guess}}^{2}=0.05)$ and 0.92 ($h_{\text{guess}}^{2}=0.30$), and decreased as υ increased. The lowest correlation betwenn GBLUP and TMAP was 0.965; the value of the metric increased with ν and with $h_{\text{guess}}^{2}$. Even though correlations (ρ) were large (as it is most often the case when prediction models are compared), it must be kept in mind that estimates of ρ can be driven by extreme points. Figure 3 further indicated that scatter near the middle of the distribution was appreciable. If cows were selected toward some intermediate optimum, perhaps the method of evaluation could impact selection decisions more markedly than selection for extremes.

There was no evidence of overfit by any of the models. For example, correlations between phenotypes and fitted genetic values with GBLUP were 0.74 and 0.77 for $h_{\text{guess}}^{2}=0.05$ and 0.30, respectively. For TMAP4 (TMAP8) the corresponding correlations were 0.75 and 0.79 (0.78) and, for LMAP, estimates of ρ were 0.67, and 0.72. The lower correlations obtained with LMAP are consistent with the larger scatter and range of fitted values for LMAP (Figure 3). TMAP-4 produced a larger range than GBLUP but smaller than LMAP.

**Figure 4:**
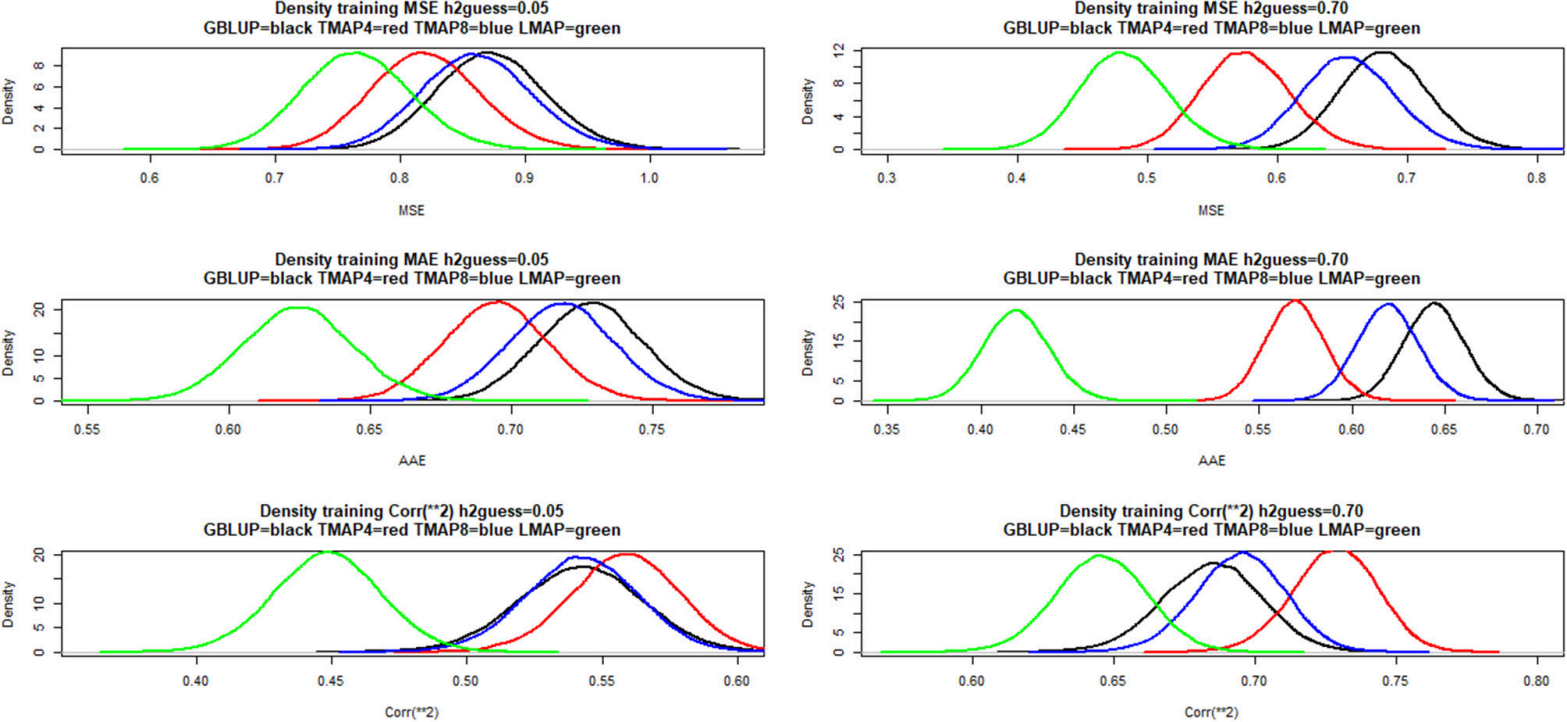
Density of training bootstrap distribution (50,000 samples) of three measures of goodness of fit of various models to milk yield test-day records in Italian Brown Swiss cows at two MINQUE “guesses” of genomic heritability. MSE: mean-squared error of fit. MAE: mean absolute error of fit. Corr(**2): squared correlation between fitted and observed phenotypes. GBLUP: genomic best linear unbiased prediction. TMAP-4 (8): maximum a posteriori with residual *t*—distribution on 4 (8) degrees of freedom. LMAP: maximum a posteriori with a double exponential residual distribution. MINQUE: minimum norm quadratic unbiased estimator of variance components.

Sampling distributions of training mean-squared error (MSEF), mean absolute error (MAE) and squared correlation between fitted values and phenotypes were examined using a simple bootstrapping scheme. Here, 50,000 samples were drawn by sampling with replacement rows from a matrix with cows in rows and phenotypes and fitted values (in columns); the metrics were computed for each of the bootstrap samples. Density estimates are shown in Figure 4 for $h_{\text{guess}}^{2}=0.05$ (left panels) and 0.70 (right panels). LMAP had the lowest MSEF and MAE, but a smaller $\rho_{\text{Fit}}^{2}$ than GBLUP or TMAP4. The density plots reaffirm (e.g., González-Recio et al., 2014) that correlation (reflecting association) and mean squared or mean absolute error (reflecting accuracy or closeness) measure different aspects of a model. Saliently, GBLUP fitted the data worse than TMAP-4 and TMAP-8 for the three end-points considered, and was also worse than LMAP in the MSE and MAE senses. However, the ability of a model to describe current data is not necessarily indicative of its predictive performance, an issue of main interest in our study.

#### 5.1.4. Leave-one out cross-validation

Generalized cross-validation (GCV) was used to locate the $h_{\text{guess}}^{2}$ expected to deliver the best predictive performance in the MSE sense of (28) and to examine sensitivity; the formulae apply to GBLUP only. The criterion was computed for each of the 19 $h_{\text{guess}}^{2}$ values. Guesses of genomic heritability of 0.15 and 0.20 seemed adequate for attaining the best possible predictive MSE; such guesses corresponded to MINQUE estimates of $h_{g}^{2}$ of 0.09 and 0.10, which are not far from the maximum likelihood estimate of 0.07. As noted by Thompson (2001) a cross-validatory approach may suggest a different value of a regularization parameter than the one attained by using likelihood or Bayesian methods applied to the entire sample available.

Next, we carried out a leave-one-out (LOO) cross-validation, a typically conservative setting (Seber and Lee, 2003; Gianola and Schön, 2016) that is widely applied in carefully and extensively controlled and measured experiments resulting in small samples, e.g., *Drosophila*, *Arabidopsis* or *Caenorhabditis* genome projects. The same grid of $h_{\text{guess}}^{2}$ was used in LOO but, instead of running 991 implementations, formula in Gianola and Schön (2016) were employed to obtain LOO metrics indirectly from the analysis of the entire sample of cows. Parameter λ was evaluated at the MINQUE estimates obtained with the entire data set, as it was reasonable to expect that removal of a single data point would not have appreciable effects on variance component estimates. Briefly, note that GBLUP, TMAP or LMAP have the general form **C**^−1^**y**,where **C** = [**I**+*s***P**]^−1^. In GBLUP *s* = λ and **P** = **K**^−1^;in TMAP *s* = λ^′′^ and **P** = (**KD**)^−1^; in LMAP *s* = ω and **P** = (**KM**)^−1^. Under this representation, the MAP LOO estimate of the additive genomic value of cow *i* can be calculated for any of the three methods as

(29) $${\hat{g}}_{i,LOO}\left(h_{\text{guess}}^{2}\right)=\frac{{\hat{g}}_{i}-c^{ii}y_{i}}{1-c^{ii}},$$

where ${\hat{g}}_{i}$ is the estimate obtained with the entire data set and *c*^*ii*^ is the *i*th diagonal element of **C**^−1^. The LOO predictive mean-squared error is

(30) $$PMSE_{LOO}(h_{\text{guess}}^{2})=\frac{{\left(y-{\hat{g}}_{LOO}\right)}^{\prime }\left(y-{\hat{g}}_{LOO}\right)}{n}.$$

Predictive correlations between ${\hat{g}}_{i,LOO}$ and *y*_*i*_ and the regression of *y*_*i*_ on ${\hat{g}}_{i,LOO}$ were calculated as well; the intercept (α) and slope (β) of such regressions can be viewed as measures of prediction bias: an “unbiased” prediction machine would be expected to produce a null intercept and a slope equal to 1. However, α and β may be near 0 and 1 even when there is a non-linear relationship between predictions and targets; as for the correlation, a straight line fit can be driven by extreme data points.

**Figure 5:**
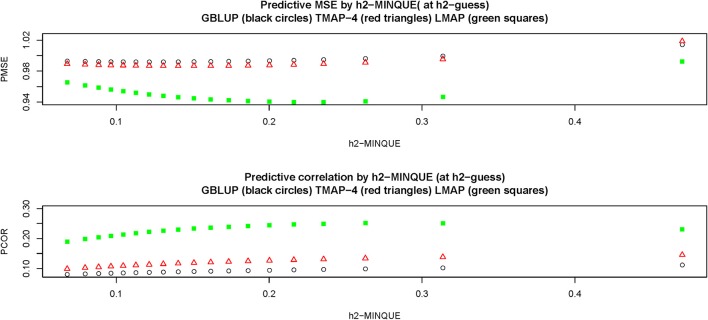
Leave-one-out cross-validation predictive mean-squared error (PMSE) and predictive correlation (PCOR) at each of 15 MINQUE guesses of genomic heritability (0.05–0.95, increments of 0.05). GBLUP: genomic best linear unbiased prediction. TMAP-4: maximum a posteriori with residual *t*—distribution on 4 degrees of freedom. LMAP: maximum a posteriori with a double exponential residual distribution. MINQUE: minimum norm quadratic unbiased estimator of variance components.

Figure 5 depicts predictive LOO MSE (PMSE) and predictive correlations (PCOR) at each of the $h_{\text{Minque}}^{2}$ estimates resulting from the $h_{\text{guess}}^{2}$ levels in the grid. Clearly, LMAP had the best predictive performance throughout whereas GBLUP had the smallest predictive correlation and typically the largest MSE. Differences between GBLUP and TMAP were small, but TMAP-4 had the second best predictive performance. LMAP behaved differently from GBLUP and TMAP with respect to the strength of regularization, illustrating that the guideline (MSE sense) prescribed by GCV was reasonable for GBLUP and perhaps TMAP, but not for LMAP. For example, PMSE decreased in LMAP until $h_{\text{Minque}}^{2}$ was 0.19 (corresponding to $h_{\text{guess}}^{2}=0.65$) and then started to increase, and PCOR increased monotonically until $h_{\text{Minque}}^{2}=0.31$ (for $h_{\text{guess}}^{2}=0.90$). Predictive association (measured as correlation) and closeness or accuracy of predictions were not proxies for each other, as observed by Gianola (2017).

**Figure 6:**
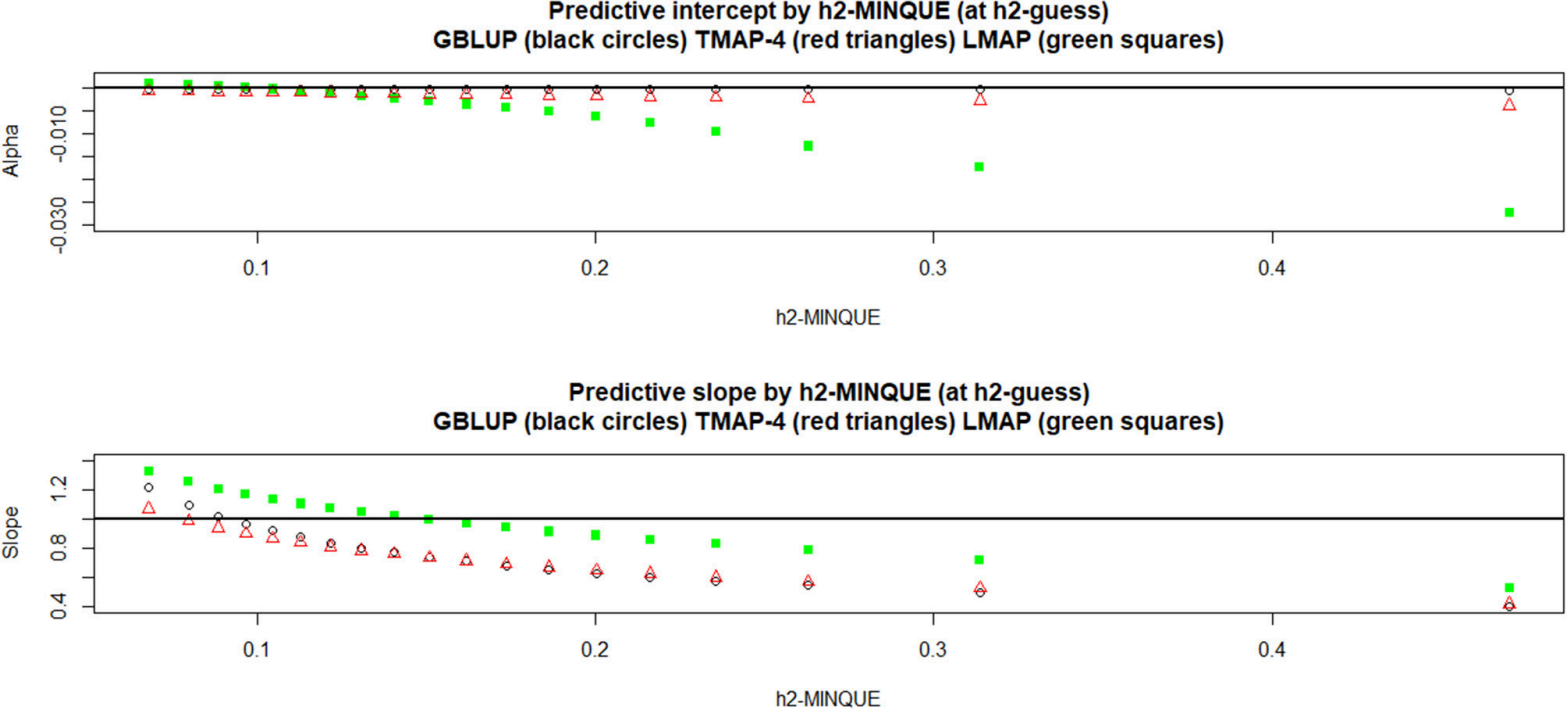
Leave-one-out cross-validation predictive intercept (Alpha) and slope (Beta) of the regression of phenotype on prediction at each of 15 MINQUE guesses of genomic heritability (1: 0.05, 2: 0.10,…, 15: 0.95). GBLUP: genomic best linear unbiased prediction. TMAP-4 (8, 12, 16): maximum a posteriori with residual *t*—distribution on 4 (8, 12, 16) degrees of freedom. LMAP: maximum a posteriori with a double exponential residual distribution. MINQUE: minimum norm quadratic unbiased estimator of variance components.

Figure 6 displays the behavior of the estimates of predictive α and β. Regularization affected “bias”: for instance, GBLUP and TMAP-4 produced estimates of α practically not departing from 0, whereas LMAP was “on target” until $h_{\text{Minque}}^{2}$ was slightly larger than 0.10, but had a clear downwards bias thereafter. Estimates of β were near 1 for GBLUP and TMAP-4 when $h_{\text{Minque}}^{2}$ was about 0.10, but had a downwards bias as heritability increased further. For LMAP, the “bias” for β was smaller than for either GBLUP and TMAP at the heritabilty levels where predictive performance was best in the MSE sense, i.e., $h_{\text{Minque}}^{2}\approx 0.20$. In a nutshell, no method was uniformly best for all four metrics used (PMSE, PCOR, α and β),making patent that goodness of predictions and “unbiasedness” do not necessarily align, a result that is consistent with literature in statistical and machine learning (e.g., James et al., 2013).

**Figure 7:**
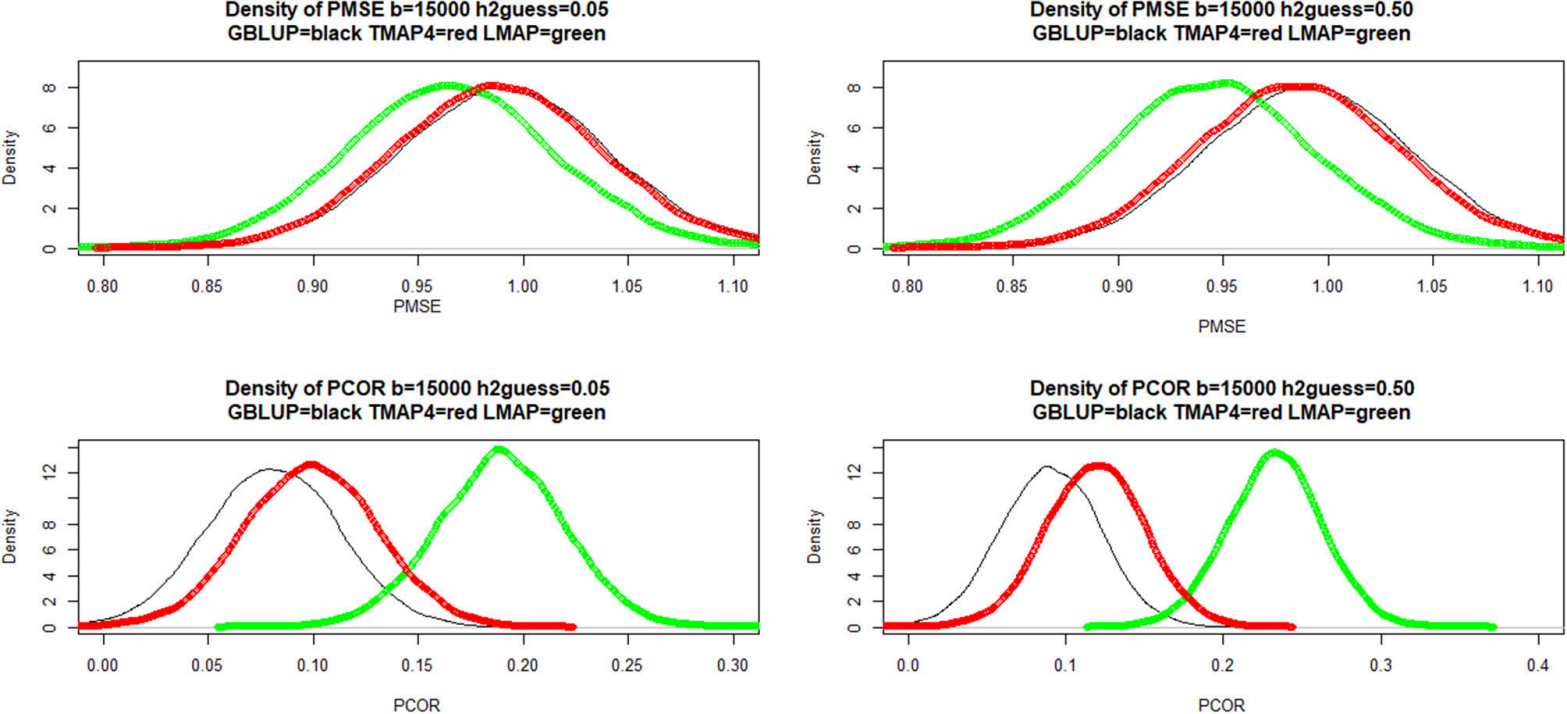
Density of bootstrap distribution (15,000 samples) of leave-one-out cross- validation predictive mean-squared error (PMSE) and predictive correlation (PCOR) at two MINQUE guesses of genomic heritability. GBLUP: genomic best linear unbiased prediction. TMAP-4: maximum a posteriori with residual *t*—distribution on 4 degrees of freedom. LMAP: maximum a posteriori with a double exponential residual distribution. MINQUE: minimum norm quadratic unbiased estimator of variance components.

Density estimates were obtained from 15,000 bootstrap samples of the LOO cross-validation, conditionally on the training set; these densities are presented in Figure 7 (only TMAP-4 is shown because TMAP-8-TMAP-16 were similar to GBLUP). For PMSE there was some overlap between densities (especially between GBLUP and TMAP-4) but LMAP appeared better at both $h_{\text{guess}}^{2}=0.05$ and 0.50. For PCOR the best performance was attained by LMAP followed by TMAP-4 and then by GBLUP. In short, LMAP appeared as the best prediction machine for this data set when using these metrics, but at the expense of some empirical bias when PMSE was smallest and PCOR largest.

Reviewer 2 suggested that consideration be given to an information retrieval measure (Blondel et al., 2015; Järvelin and Kekäläinen, 2017, Ma et al., in review) for comparing the various procedures. In our context, the “highly relevant” items in a search of items might be the targets having the “best” phenotypes or true breeding values (if observed, which is never the case), and the “retrieval score” is the prediction produced by any of the methods entertained. The “best” items are deemed to be more valuable than those down in an ordered list of targets, so a discount factor is introduced, discounting members of a pair as one moves from the top to the bottom of a list of graded items (cows in our case). We employed a metric called “mean normalized discounted cumulative gain” (MNDCG) as implemented in the R package DeepGS (Ma et al., in review). After adding a constant to targets and to predictions, in order to produce positive numbers, the MNDCG ranges between 0 and 1, with values closer to 1 indicating higher ability of a prediction machine to identify predictands deemed as most valuable (e.g., individuals with top breeding values), as suggested by Blondel et al. (2015). Table 1 shows that GBLUP had the worst performance with respect to the MNDCG metric applied to the top 5, 10, 20 and 50 ranks, and LMAP had the best performance, followed by TMAP-4.

**Table 1:** Mean normalized discounted cumulative gain (MNDCG) values for various methods of genome-enabled prediction of test-day milk yield in Brown Swiss cattle, at two levels of genomic heritability guess (0.30 and 0.70).

	**GBLUP**	**LMAP**	**TMAP4**	**TMAP8**	**TMAP12**	**TMAP16**
***h***${}_{g}^{2}$=**0.30**						
*k*=5	0.01	0.23	0.12	0.08	0.08	0.07
*k*=10	0.02	0.24	0.10	0.08	0.08	0.07
*k*=20	0.05	0.24	0.12	0.10	0.10	0.09
*k*=50	0.08	0.23	0.13	0.11	0.11	0.10
***h***${}_{g}^{2}$=**0.70**						
*k*=5	0.06	0.26	0.13	0.11	0.10	0.10
*k*=10	0.06	0.26	0.12	0.12	0.09	0.09
*k*=20	0.08	0.27	0.13	0.13	0.11	0.10
*k*=50	0.10	0.26	0.14	0.14	0.12	0.12

*Methods are GBLUP, LMAP^1^, TMAP4^2^, TMAP8^2^, TMAP12^2^, and TMAP16^2^. k = 5, 10, 20, 50 is the number of top ranked individuals at which MNDCG is calculated. The MNDCG criterion has a value equal to 1 when a prediction is perfect (the ranks of predictors and predictands are identical), provided predictor and predictand are positive*.

1 LMAP, Laplace's maximum a posteriori

2 *TMAP4–8-12-16: t—distribution on 4, 8, 12, 16 degrees of freedom*.

### 5.2. Arabidopsis

#### 5.2.1. Descriptive aspects

QQ plots (Figure S3) suggested that a normal process did not fit adequately any of the three phenotypes, but more appreciably flowering time and FRIGIDA expression. Maximum likelihood (MINQUE) estimates of genomic heritability were 0.9186 (0.9187) for flowering time; 0.4738 (0.4739) for FRIGIDA, and 0.4864 (0.4864) for plant diameter. In spite of departures from normality, maximum likelihood (with Gaussian assumptions) and MINQUE estimates were similar, which is remarkable in view of the small sample sizes (*n* =194, 164, and 180 for the three traits above, respectively). Genomic heritability near 1 for flowering time may be due to existence of genes (unknown) of major effects on the trait (Chiang et al., 2009). Salomé et al. (2011) studied 18 *Arabidopsis* accessions and 17 derived F2 populations and concluded that much of the variation in flowering time appeared to be due to large-effect mutations.

#### 5.2.2. Leave-one out cross-validation

Models (zero-mean) fitted were GBLUP, LMAP and TMAP with υ = 4, 8, 12, 16, or 20. Analyses with the entire data set indicated that TMAP converged in less than 15 iterations. LMAP needed additional rounds but, after 50–100 iterations, the average *m*−value was changing after the second-third decimal place only. As expected, average *d*−values in TMAP increased with ν,although the pattern was not monotonic for flowering time, a trait for which major genes underlie variation, as noted. Average *m*−values in LMAP were much larger for flowering time than for FRIGIDA expression or plant diameter, suggesting better fit of the *DE* model for the former trait.

Correlations between phenotypes and fitted values from all methods using the entire data set were nearly perfect for flowering time, ranged between 0.93 (TMAP-4) and 0.94 (TMAP-8) for FRIGIDA, and between 0.92 (GBLUP) and 0.96 (TMAP-8) for plant diameter. The correlations suggested over-fitting especially for flowering time. Inter-correlations between predictions were close to 1 for flowering time and were not lower than 0.93 for the other two traits.

Given the small *n*,removal of a single datum could have a marked impact on estimates of variance components so it would have not been reasonable to use formulae in Gianola and Schön (2016) that assume constancy of regularization parameters in LOO. Therefore, we carried out a “brute force” LOO cross-validation by re-estimating variance components by MINQUE (ML estimates from the overall data used as “guess”) at each training instance; e.g., for flowering time there were 194 sets of MINQUE estimates. At any such instance, whenever a negative MINQUE estimate of a variance component was encountered (it happened only once for the residual variance of flowering time), it was replaced by the ML estimate found with the entire data set. The range of LOO estimates of $h_{g}^{2}$ was 0.8979–0.9326 (flowering time), 0.3925–0.7307 (FRIGIDA) and 0.4523–0.5930 (diameter), so removal of a single observation was indeed influential in regularization, especially for FRIGIDA. In the light of what had been found with the entire data set, all LOO cross-validation implementations of LMAP and TMAP were run for a conservative 200 iterations, assuming convergence at that point. For each trait, a matrix was formed by column-wise concatenation of phenotypes and of their corresponding predictions. The *n* rows of each of these matrices were re-sampled with replacement *b* = 50, 000 times to construct bootstrap distributions of PMSE, PCOR and of the intercept (α) and slope (β) of the regression of targets on predictions. These distributions describe uncertainty, conditionally on the training set.

**Figure 8:**
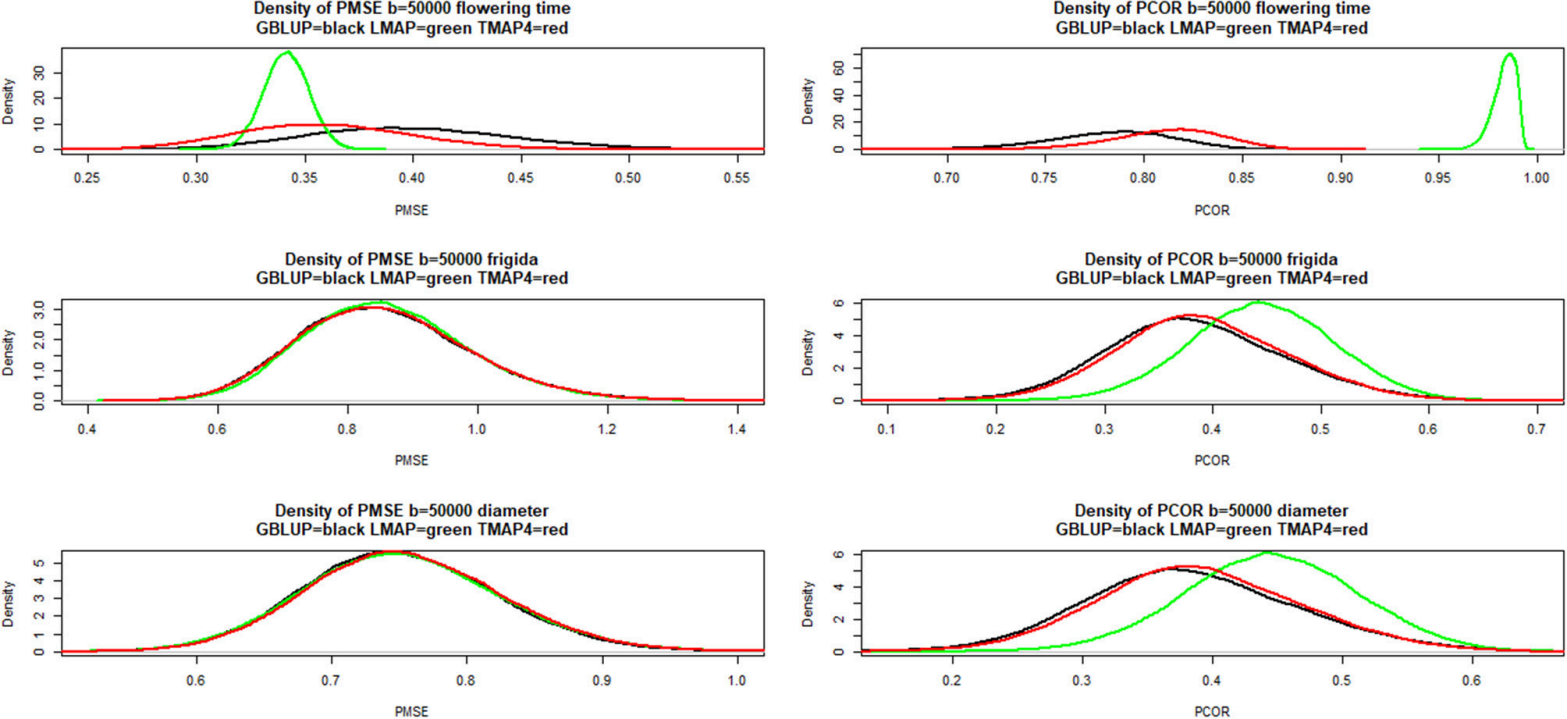
Density of bootstrap distribution (50,000 samples) of leave-one-out cross- validation predictive mean-squared error (PMSE) and predictive correlation (PCOR) of flowering time, FRIGIDA gene expression and plant diameter in Arabidopsis. GBLUP: genomic best linear unbiased prediction. TMAP-4: maximum a posteriori with residual *t*—distribution on 4 degrees of freedom. LMAP: maximum a posteriori with a double exponential residual distribution.

Figure 8 presents estimates of the density of bootstrap distributions of PMSE (left panels) and PCOR (right panels), respectively, for the three “most different” methods (GBLUP, LMAP, TMAP with ν = 4) for each of the three traits. Flowering time was the only trait for which a marked difference was observed (LMAP being the most different from GBLUP). For this trait, LMAP displayed the smallest PMSE and the largest PCOR; bootstrap distributions for LMAP were clearly sharper than for other procedures. The second best method for flowering time was TMAP4. For FRIGIDA and plant diameter the distributions overlapped considerably with LMAP slightly better as per the PCOR criterion.

**Figure 9:**
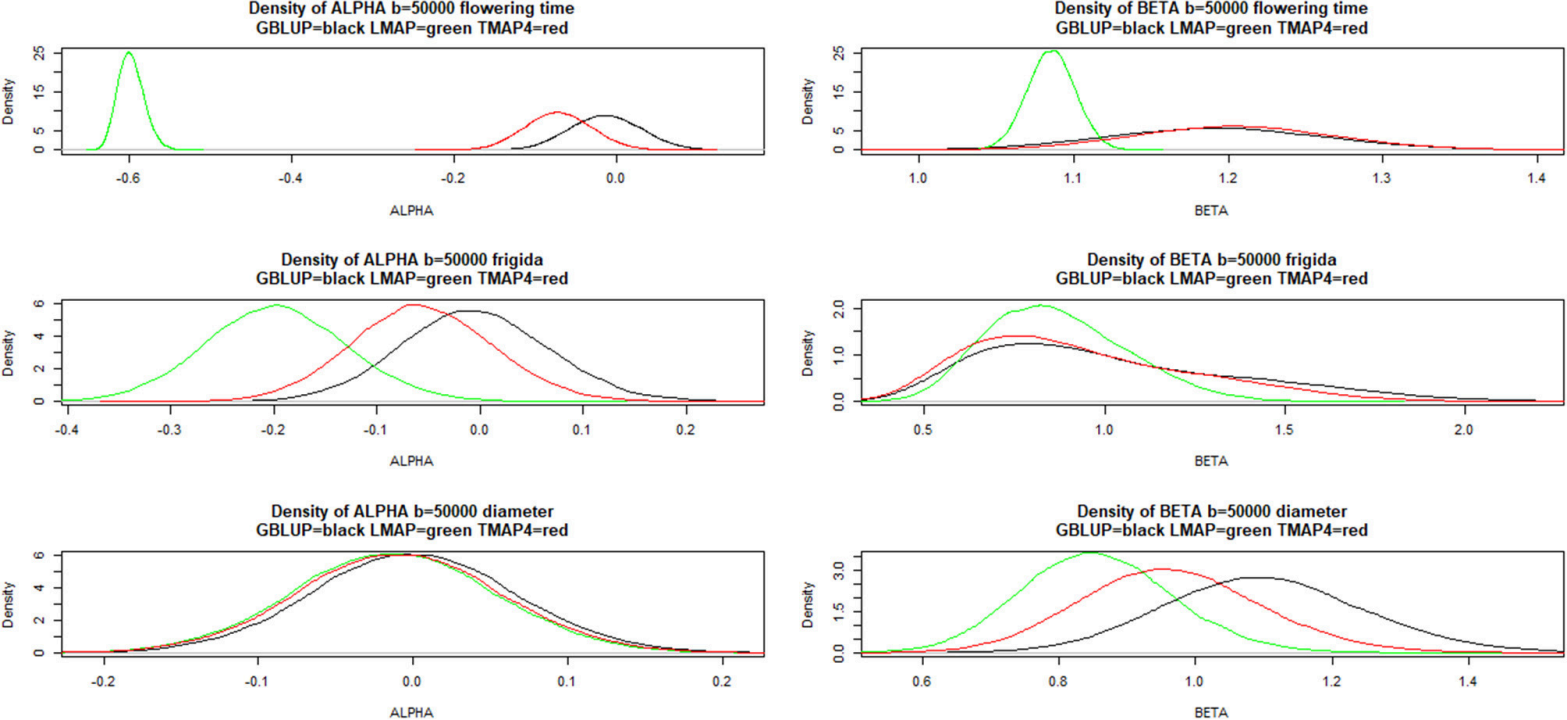
Density of bootstrap distribution (50,000 samples) of leave-one-out cross- validation predictive intercept (ALPHA) and predictive slope (BETA) of the regression of phenotype on prediction for flowering time, FRIGIDA gene expression and plant diameter in Arabidopsis. GBLUP: genomic best linear unbiased prediction. TMAP-4: maximum a posteriori with residual *t*—distribution on 4 degrees of freedom. LMAP: maximum a posteriori with a double exponential residual distribution.

Figure 9 shows that LMAP was both intercept and slope “biased” for flowering time, whereas α = 0 was assigned appreciable density in the bootstrap distributions of the other two methods; slopes were “biased” upwardly for all three methods. For FRIGIDA and plant diameter, bootstrap distributions of estimates of α and β overlapped some for all models, and evidence of departure from 0 and 1 was not as compelling as for flowering time.

A desirable property of a prediction machine is exhibiting a consistent performance over many samples. Our bootstrapping emulated random sampling from the distribution of testing set (conditionally on the training set), so it provided a means for evaluating consistency of predictions. Since we run all methods in each of the samples, paired comparisons, more precise than gross comparisons, could be carried out. The outcome of paired comparisons of GBLUP vs. the six thick-tailed models fitted is given in Table 2. The entries of the table are the relative frequencies (over the 50,000 bootstrap samples) with which GBLUP had either a smaller PMSE or a larger PCOR than LMAP or any of the TMAP models. For flowering time, LMAP gave a smaller PMSE than GBLUP in 87% of the samples and had a larger PCOR 100% of the time (it also had a larger PCOR than any of the TMAP specifications in 100% of the samples, not shown here). For FRIGIDA, PMSE for GBLUP was smaller in 54% (LMAP) and 53% (TMAP-4) of the samples; however, TMAP8-12-16-20 had a better performance than GBLUP about 65% of the time. PCOR in FRIGIDA was larger for GBLUP than for LMAP only 6% of the time, and in 27–33% of the draws when compared with the *t*-models. For plant diameter, GBLUP was better most of the time, save for PCOR when compared with LMAP.

**Table 2:** Fraction of bootstrap samples (50,000) in which genomic best linear unbiased prediction (GBLUP) attained a smaller prediction mean-squared error (PMSE) or a larger predictive correlation (PCOR) than either LMAP^1^, TMAP4^2^, TMAP8^2^, TMAP12^2^, TMAP16^2^, or TMAP20^2^.

	**GBLUP vs. LMAP**	**GBLUP vs. TMAP4**	**GBLUP vs. TMAP8**	**GBLUP vs. TMAP12**	**GBLUP vs. TMAP16**	**GBLUP vs. TMAP20**
**FLOW**						
PMSE	0.13	0	0	0	0.00	0.00
PCOR	0	0	0	0	0	0
**FRIG**						
PMSE	0.54	0.53	0.33	0.35	0.36	0.37
PCOR	0.06	0.30	0.27	0.29	0.31	0.33
**DIAM**						
PMSE	0.52	0.65	0.59	0.57	0.58	0.55
PCOR	0.39	0.81	0.82	0.81	0.80	0.80

*Target traits are flowering time (FLOW), FRIGIDA gene expression (FRIG) and plant diameter (DIAM) in Arabidopsis thaliana*.

1 LMAP, Laplace's maximum a posteriori

2 *TMAP4–8-12-16-20: t—distribution on 4, 8, 12, 16, or 20 degrees of freedom*.

These findings indicated (most notably for flowering time and to a lesser extent for FRIGIDA) that a robust residual distribution may deliver better predictions than GBLUP most of the time for some traits, even though the procedures are not unbiased, neither theoretically nor empirically. Results for flowering time suggest that, when unknown major genes are suspected to underlie genetic variation and an “infinitesimal genomic” model is fitted, use of a thick-tailed residual distribution protects against model mis-specification and produces better predictions at a lower level of uncertainty (sharper bootstrap distributions). On the other hand, when trait residuals conform close but not perfectly to a Gaussian distribution, such as in the case of plant diameter, a robust method can yield an equivalent and, sometimes, a better performance than GBLUP, as seen in the case of the PCOR metric.

### 5.3. Wheat

Grain yield in cereals is a multifactorial trait affected by non-additive gene effects, strong environmental forces and genotype by environment interaction (Singh et al., 1986; Sleper and Poehlman, 2006; Huang et al., 2010). Further, the wheat data set employed here was found (Gianola et al., 2016) to have a complex underlying sub-structure, e.g., a multi-dimensional scaling analysis of a genomic relationship matrix constructed with 1,279 markers indicated that the first coordinate separated the *n* = 599 lines into two groups whereas the second coordinate stretched lines over the y-axis. Janss et al. (2012) argued (from a random effects model perspective) that use of **G** automatically corrects for substructure, but there is no general agreement on the matter. If such heterogeneity were associated with variation in mean values, a simple additive zero-mean Gaussian model fitted to grain yield could still produce outliers because marker effects would be shrunk to a common mean, thus ignoring existence of strata with potentially different means (as in “grouping” of ancestors in animal breeding).

The predictand chosen was wheat grain yield with all 599 lines planted in four distinct environments; the corresponding vectors of phenotypes are denoted as **y**_1_, **y**_2_, **y**_3_, and **y**_4_. We also formed “composite” traits by summing pairs, triplets and tetraplets of yields in different environments, i.e., **y**_1+2_, **y**_1+3_,…, **y**_1+2+3_,…, **y**_2+3+4_, **y**_1+2+3+4_,so the total number of response variables was 15. For the “yield sums,” the expectation was that their distribution would be more nearly Gaussian than those of elementary yields, thus providing a challenge to the thick-tailed models in situations in which normality probably holds. QQ plots for the 15 yield traits are in Figures S4–S7; the closest conformity with the Gaussian assumption seemed to be for sums of pairs of yields, but no improvement in fit was observed for triplets or for the tetraplet yield.

A training (*n* = 300)-testing (*n* = 299) layout was repeated 200 times, completely at random. At each training instance, variance components were estimated by MINQUE using maximum-likelihood estimates as guesses; models fitted were BLUP, LMAP and TMAP (ν =4, 6, or 8). Analyses were done using pedigree (**A**) or genomic kinship (**G**) matrices.

**Figure 10:**
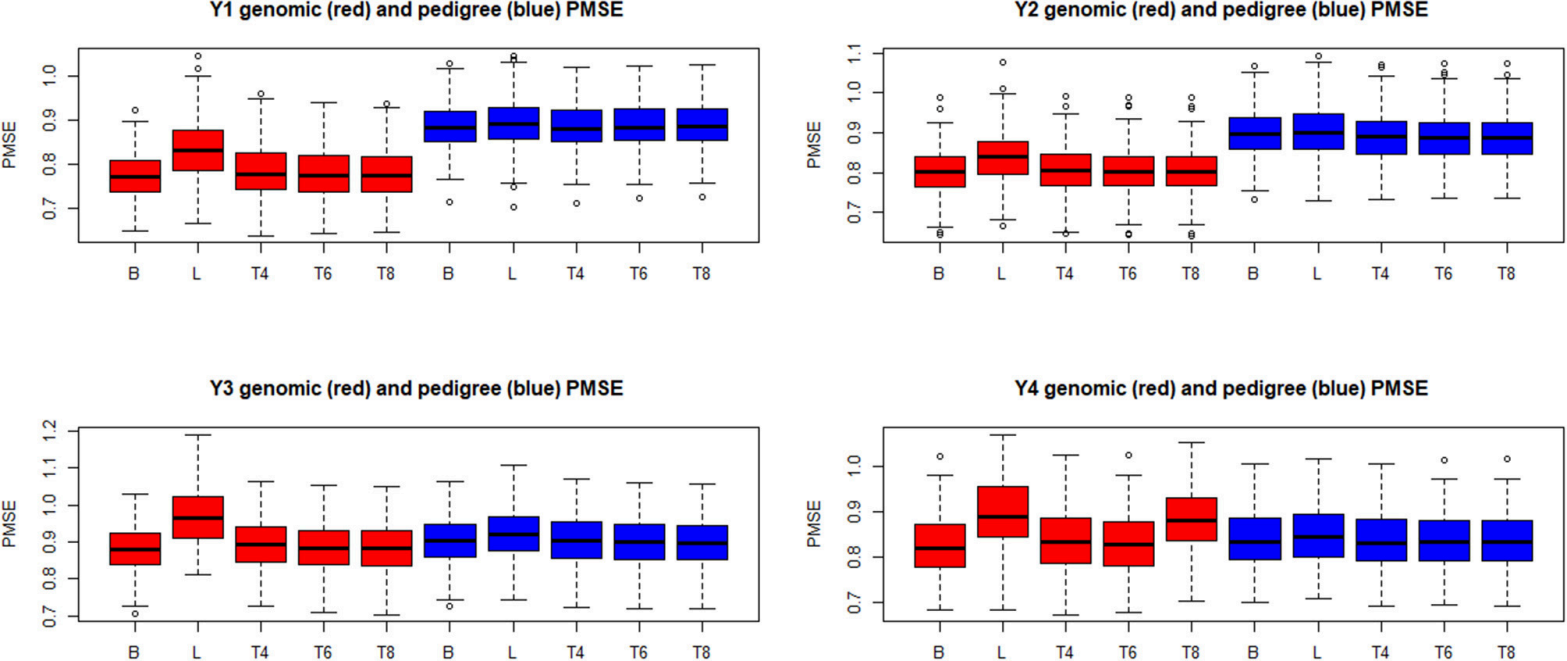
Box plots of the distribution (200 randomly reconstructed training-testing layouts) of predictive mean-squared error (PMSE) of wheat yield in four different environments (Y1, Y2, Y3, Y4). B: best linear unbiased prediction. L: maximum a posteriori with a double exponential residual distribution. T4, T6, T8: maximum a posteriori with residual *t*—distribution on 4, 6, or 8 degrees of freedom, respectively.

**Figure 11:**
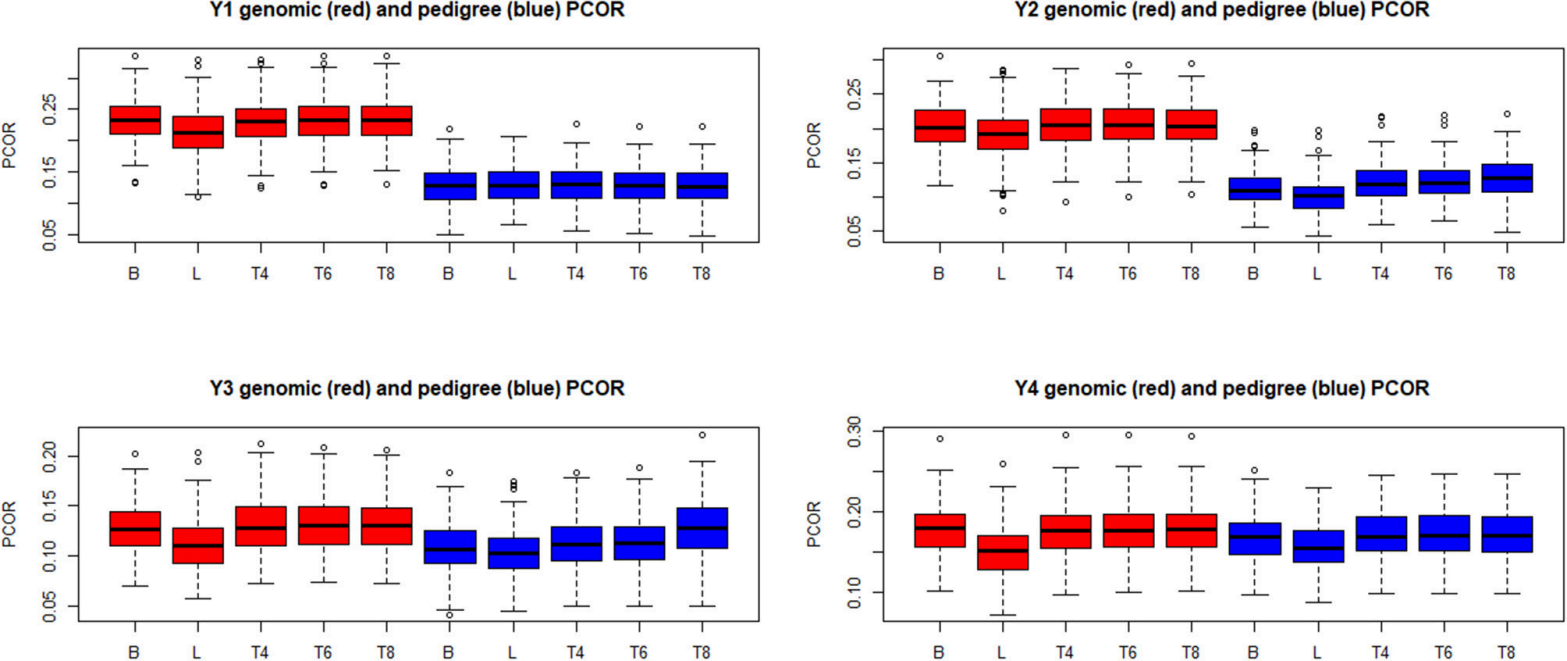
Box plots of the distribution (200 randomly reconstructed training-testing layouts) of predictive correlation (PCOR) of wheat yield in four different environments (Y1, Y2, Y3, Y4). B: best linear unbiased prediction. L: maximum a posteriori with a double exponential residual distribution. T4, T6, T8: maximum a posteriori with residual *t*—distribution on 4, 6, or 8 degrees of freedom, respectively.

Boxplots of the distributions of PMSE and PCOR are in Figures 10, 11, respectively, for each of the four “elementary” yields. Predictions based on **G** were better than those obtained with **A** for yields 1 and 2, but not for **y**_3_ and **y**_4_. Within environment and type of kinship matrix, the five models did not differ by much in predictive performance, especially in the light of the large variability among replicates. The DE model (L in the figures) tender to deliver larger PMSE and smaller PCOR than other methods.

We also examined consistency of predictive performance over the 200 replications by calculating the frequency with which a given method delivered the best predictions. Tables 3, 4 present results for PCOR when predictions for the 15 yield traits were either pedigree-based (e.g., ABLUP) or genome-based (e.g., GLMAP), respectively. For **A**-based predictions (Table 3), BLUP had the largest frequency of appearing as the best method in only in 1 of the 15 comparisons (yield trait 10). LMAP was best in 2 out of 15, and TMAP8 had the largest frequency of attaining the top PCOR in 8 such evaluations. Summing yields did not ameliorate the performance of BLUP relative to the robust methods.

**Table 3:** Frequency with which a given method had the largest predictive correlation for wheat grain yield over 200 replications of a training-testing layout: pedigree (A) based model, (“winning” method in boldface).

**YIELD TRAIT**	**ABLUP^1^**	**ALMAP^2^**	**ATMAP4^3^**	**ATMAP6^4^**	**ATMAP8^5^**
1	0.230	**0.390**	0.225	0.045	0.110
2	0.085	0.025	0.185	0.185	**0.520**
3	0.115	0.105	0.230	0.220	**0.330**
4	0.190	0.100	0.280	0.130	**0.300**
5 (1+2)	0.265	0.045	0.105	0.060	**0.525**
6 (1+3)	0.185	**0.460**	0.170	0.060	0.125
7 (1+4)	0.160	0.185	**0.320**	0.095	0.240
8 (2+3)	0.175	0.055	0.130	**0.355**	0.285
9 (2+4)	0.130	0.025	0.250	**0.310**	0.285
10 (3+4)	**0.290**	0.035	0.105	0.255	0.315
11 (1+2+3)	0.130	0.170	0.170	0.180	**0.350**
12 (1+2+4)	0.115	0.060	0.155	0.140	**0.530**
13 (1+3+4)	0.135	0.110	0.235	0.160	**0.360**
14 (2+3+4)	0.185	0.030	0.140	**0.350**	0.295
15 (1+2+3+4)	0.145	0.055	0.150	0.255	**0.395**

1 *ABLUP: best linear unbiased prediction*.

2 *ALMAP: Laplace's maximum a posteriori*.

3 *ATMAP4: t—distribution on 4 degrees of freedom maximum a posteriori*.

4 *ATMAP6: t—distribution on 6 degrees of freedom maximum a posteriori*.

5 *ATMAP8: t—distribution on 8 degrees of freedom maximum a posteriori*.

**Table 4:** Frequency with which a given method had the largest predictive correlation for wheat grain yield over 200 replications of a training-testing layout: genome (G) based model, (“winning” method in boldface).

**YIELD TRAIT**	**GBLUP**	**GLMAP**	**GTMAP4**	**GTMAP6**	**GTMAP8**
1	**0.505**	0.060	0.220	0.065	0.150
2	**0.300**	0.175	0.245	0.125	0.155
3	0.260	0.075	0.175	0.135	**0.355**
4	**0.465**	0.015	0.245	0.065	0.210
5 (1+2)	**0.460**	0.045	0.255	0.100	0.140
6 (1+3)	**0.550**	0.055	0.175	0.065	0.155
7 (1+4)	**0.460**	0.030	0.220	0.080	0.210
8 (2+3)	**0.345**	0.105	0.155	0.175	0.220
9 (2+4)	**0.330**	0.095	0.170	0.185	0.220
10 (3+4)	**0.510**	0.030	0.090	0.110	0.260
11 (1+2+3)	**0.495**	0.070	0.190	0.085	0.160
12 (1+2+4)	**0.560**	0.025	0.190	0.075	0.150
13 (1+3+4)	**0.730**	0.020	0.085	0.025	0.140
14 (2+3+4)	**0.450**	0.075	0.125	0.105	0.245

1 *GBLUP: best linear unbiased prediction*.

2 *GLMAP: Laplace's maximum a posteriori*.

3 *GTMAP4: t—distribution on 4 degrees of freedom maximum a posteriori*.

4 *GTMAP6: t—distribution on 6 degrees of freedom maximum a posteriori*.

5 *GTMAP8: t—distribution on 8 degrees of freedom maximum a posteriori*.

For genome-based predictions (Table 4), BLUP was the most frequent “winner” (14 of the 15 traits), notably for sums of yields; for example, for yield trait 13 GBLUP produced the largest PCOR in 73% of the 200 replications); for the 4 elementary yields, BLUP was best in 50.5, 30, 26, and 46.5% of the replications, respectively. We conjecture that when pedigree is used, the robust distributions mitigate somehow the impact of genomic sub-structure, strong in this wheat ensemble of lines. When markers are used for constructing the kinship matrix, this sub-structure is partially accounted for (Janss et al., 2012), perhaps rendering the Gaussian assumption less vulnerable to the effect on means of ignoring population stratification.

## 6. Discussion

Best linear unbiased prediction (pedigree or genome-based) is routinely employed for genetic evaluation of candidates for selection in breeding of crops and livestock. The method has many attractive features, such as flexibility, because it can be used in longitudinal, cross-sectional, incomplete and missing data situations and also extends to multivariate problems. The structure of the linear model is easy to amend, e.g., adding or removing fixed and random effects, and computations are deterministic, although large implementations require advanced iterative numerical methods. There is publicly available software for massive amounts of data, such as DMU (Madsen and Jensen, 2013).

In the era of genome-enabled prediction, a plethora of Bayesian linear regression models have emerged (Meuwissen et al., 2001; Gianola et al., 2009; de los Campos et al., 2013; Gianola, 2013). These Bayesian models proposed differ mainly in the specification of the prior distribution assigned to regression coefficients, so observed variation in performance is due to the prior assumptions, as expected from Bayesian theory for finite samples; the influence of priors on inference is exacerbated further by the “large *p*,small *n*” problem encountered with massive genomic data. Further, Bayesian MCMC methods require very careful calibration, some do not extend easily to multivariate situations (e.g., the double exponential prior in the Bayesian Lasso), contain Monte Carlo error and sometimes experiment difficulties in converging to the equilibrium distribution. Quite often, results may “look good” even when convergence has not been attained. In view of the preceding considerations it is not surprising that ABLUP and GBLUP continue being methods of choice for prediction of complex traits, as exemplified by it use in national and international evaluations of dairy cattle (Weigel et al., 2017).

All BLUP models, as well as Bayesian specifications, implicitly or explicitly pose a Gaussian distribution for the residuals, a process known to be sensitive to outliers and whose presence is pervasive in observational (as opposed to experimental) data. Outlying observations are typically due to missing covariables such as information on preferential treatment of animals, or ignoring presence of unknown segregating QTL and genotype × environment interactions. In the absence of a correct specification, the neglected effects are lumped most often into the model residual. The standard Gaussian assumption makes the regression function incapable of recognizing aberrant observations, unless outliers are removed *ex* *ante* or *ex* *post*. On the other hand, some thick-tailed residual distributions can recognize and differentially mitigate the impact of outliers via an automatic attenuation of phenotypes associated with the corresponding data points. The thick-tailed models, such as those based on the *t* or Laplace's distribution, confer differential weights to phenotypes and provide model-derived diagnostics of sources of discrepancy with outcomes.

In this paper we used a certain Bayesian logic, i.e., MAP instead of MCMC, for incorporating thick-tailed residual distributions while retaining a BLUP-type computational framework. The methods, which are non-linear, use re-iteration of linear mixed-model equations and can be viewed as belonging to the class of quantitative genetics generalized linear models (e.g., Thompson, 1979; Gianola and Foulley, 1983). Since TMAP and LMAP are not linear functions of the data predictions cannot be claimed to be unbiased or “best” in the standard or idealized senses. A predictive (as opposed to inferential) approach was employed here for tuning regularization parameters for these models by adopting a combination of grid search and non-iterative variance component estimation via MINQUE.

The study focused on conceptual rather than on computing matters: our primitive codes were not optimized in any sense of the word. Since reviewers expressed interest in knowing computing times, some information is provided hereby. With the cow data set (*n* = 991),the following clock time was needed for the following: (1) computing all GBLUP in the heritability grid with 15 entries: 8.51 s. (2) Ten iterations of TMAP-4 for a single heritability value: 10.82 s; (3) 30 iterations of LMAP for a single heritability entry: 49.02 s. All computations were run using a mundane laptop computer under the Windows 10 64-bit operating system; the dual processor employed was Intel (R) Core (TM() i7-5500U CPU @2.40 GHz and 2.39 GHz, and installed memory was 12.0 GB. Most calculations were completed in a few minutes of clock time, with the exception of the wheat study where, in each of the 200 replications, five different models (some iterated for 200 rounds) were fitted to each of 15 traits. Such calculation was completed overnight. A rough rule of thumb is that, if GBLUP requires *x* hours, TMAP would probably take 20*x*,and LMAP probably 100*x*. For comparison, a Bayesian MCMM method using a Gibbs sampler would probably require at least thousands of *x* for the thick tailed-methods, assuming that such an amount of sampling is adequate for reaching the equilibrium distribution and attaining a small Monte Carlo error. The efficiency of our computations could be markedly improved if the code had been written by a computing expert or written in a faster language.

Reviewer 3 suggested that we discuss use of thick-tailed distributions for modeling the “genetic” part of the linear model. Bayes A and Bayes B of Meuwissen et al. (2001) actually use independent *t*−distributions for markers effects included in a regression vector **β** (at least for non-zero state in Bayes B), and the Bayesian LASSO (Park and Casella, 2008) assigns independent DE distributions to each of the regression coefficients. However, the distributions of **g** = **X**β are intractable: linear combinations of independent *t* or *DE* random variables have unknown distributions. On the other hand, assigning a multivariate *t*—distribution to the entire **g** vector with **G** as scale matrix is feasible (Strandén, 1996), but using a grid search for $h_{g}^{2}$ and ν_*g*_ since these two parameters cannot be separated in the likelihood function; on the other hand, independent univariate *t*-distributions with the same parameters could be assigned to each of the members of series of “clusters,” as in a sire model (Strandén, 1996). It is doubtful, however, that a multivariate *t*−distribution would protect against genetic outliers because the attenuation operates on the entire **g** and not on its individual elements (Strandén, 1996). Further the independent *t*−model for clusters would introduce an extra parameter in the grid: the “cluster” degrees of freedom.

Reviewer 1 encouraged us to discuss a method (called “R”) presented by Reverter et al. (1994a,b), developed with the aim of detecting “prediction bias” and estimating variance component in a mixed effects linear model. Method “R” takes the view that such model holds true, and it forces the regression of predictands on predictors to be equal to 1, implicitly produceing “empirically unbiased” predictions. Although ingenious, “R” can be deceiving: it forces predictions and parameter estimates to comform to a given model, as to opposed to searching for a specification that reflect the nature of the data, which arguably is what one ought to do in science. “R” should not be prescribed unless there is complete certainty that a mixed effects linear model provides the best prediction machine. Thompson (2001) provided a discussion of “R” and a comparison with some of the cross-validation approaches used in our paper, and wrote: “These cross-validatory techniques are useful when a prescription for prediction exists but no formal variance structure exists.” While genetic relatedness provides information about part of the “variance structure,” one should keep in mind that there are other issues that also deserve consideration in analysis of complex traits, e.g., skweness, kurtosis, outliers, hidden structure, non-random missing data, unknown major genes, non-additive gene action, epigenetics, etc. In short, one should not claim or believe (given the current state of knowledge) that a “formal variance structure” always holds while ignoring many other aspects of model building. Quantitative genetics (and science, more generally) goes beyond **y** = **Xβ** + **Zu** + *e*, the standard general linear model under Gaussian assumptions. Our perspective is representative of the perspective taken in the fields of statistical and machine learning, i.e., one of caution with respect to making strong assumptions concerning mechanisms generating data sets.

Using dairy cattle, *Arabidopsis* and wheat data, proof-of-concept was provided that models with *t* or Laplace residual distributions can often deliver closer (e.g., in the sense of PMSE and PCOR) predictions to targets than BLUP. In particular, we found that the robust methods outperformed GBLUP in data sets representing Brown Swiss cattle (test-day milk yield) and *Arabidopsis* (notably flowering time and FRIGIDA gene expression). Our relatively simple tuning of regularization parameters permited emulation of conceptual repeated sampling (bootstrapping) as well as replication of a training-testing layout. It was also found that the robust alternatives to BLUP were often more stable over conceptual repeated sampling, e.g., displaying a lower PMSE consistently.

We also verified empirically that the best predictions were not necessarily associated with “unbiasedness,” an obsession of (many) animal breeders since the introduction of BLUP. Our study focused on a univariate linear model, but the robust distributions can also be used with any non-linear model in which residuals enter additively into the model structure, e.g., growth and lactation curve models. Typically it is unreasonable to expect that non-linear predictors derived from a growth or lactation curve are unbiased, and such property is irrelevant if the objective of an analysis is to obtain the best possible predictions. Although the “best predictor” (conditional mean) in the mean squared error sense is unbiased, the property holds only if the joint distribution of random effects (e.g., genotypic values) and of phenotypic values is known without error (Henderson, 1973; Fernando and Gianola, 1986). Hence, any time model assumptions are violated, the “best predictor” must give away the “yellow jersey,” a term well known by followers of the Tour de France.

Reviewer 1 expressed that the treatment of unbiasedness dispensed in the preceding paragraph was “unfair,” and argued that unbiased estimation of differences between age cohorts is needed for “correctly ascertaining” genetic trends in animal breeding, as if this were possible (true genetic trend is unknown). The current state of statistical science refutes such view: the objective of any statistical model is to get as close as possible to estimands (good “Oracle” properties in modern lingo), which is seldom accomplished by unbiased procedures. For example, the so called “James-Stein” phenomenon shows that the best linear unbiased estimator of a location vector with at least two unknown parameters is inadmissible. Judge et al. (1985) discussed the issue from an econometrics perspective, a field in which finding good estimates of parameters is no less important than in animal breeding. Further, ridge regression (a biased estimator) was developed to reduce Euclidean distance between the estimator and the true regression vector relative to what would be attained with ordinary least-squares (an unbiased procedure); see Hoerl and Kennard (1970). More recently, Meyer (2016) deliberately introduced bias (via penalty functions) in maximum likelihood estimation of variance and covariance components simply because the biased estimates can often be closer to the true values. Returning to BLUP: it is well known that in a simple model in which the regression vector is declared random, BLUP and ridge regression produce exactly the same results at the same level of regularization. Then, how can BLUP be unbiased and biased at the same time? The explanation is that animal breeders often fail to note that BLUP is unbiased with respect to the mean of the distribution of the random effects, but not with respect to their realized values in an extant data set. The problem is with the frequentist definition of BLUP and not with what the method does. In fact, one can interpret BLUP as a Bayesian estimator (biased, unless the prior were “true”); as a penalized maximum likelihood estimator (biased in small samples); or as a reproducing kernel Hilbert spaces regression (RKHS) or as a linear single-neuron network with an identity activation function. For the last two interpretations, what matters is the variance-bias trade-off, and the best settings are typically those in which some bias is accepted in order to decrease variance. In a nutshell, the importance of unbiasedness has been overstated in the field of animal breeding as clearly pointed out in many papers and textbooks, e.g., Blasco (2001, 2017).

Our approach can also be used in single-step BLUP (e.g., Legarra et al., 2009) and in RKHS (e.g., Gianola et al., 2006; Gianola and Van Kaam, 2008). For a single-step BLUP mixed effects model with *t*-residuals, for example, the iteration has the same form as Equation (7) except that **K** is replaced by a matrix **H** (involving **A** and **G** relationship matrices) that is also a function of the degree of similarity between individuals with and without marker genotypes. In the case of RKHS, the matrix **K** in, e.g., de los Campos et al. (2009) and Gianola and Schön (2016), would be replaced by a more general kernel matrix, such as a linear combination of Gaussian kernels.

As pointed out earlier, multiple-trait models can be fitted with reasonable ease in ABLUP or GBLUP with main challenges being their computation. There is a fairly widespread view that multiple-trait models can account for some forms of bias in inference of breeding values and of genetic parameters (Thompson, 1979; Gianola et al., 1988; Im et al., 1989). Multivariate outliers are more subtle and delicate than single-trait model discrepancies, so extensions of TMAP and LMAP might be useful. If a *t*−distribution is used, extension to multiple-traits with imputation of missing records is fairly straightforward. Actually, Strandén (1996) fitted additive models with univariate and bivariate residual *t*—distributions to data on milk yield of Finnish Ayrshire cattle (Bayesian MCMC was used) and found stronger support for the thick-tailed processes. TMAP for the multiple-trait case has not been developed yet, but it should not be difficult, at least conceptually (Strandén, 1996).

On the other hand, there is not much theory or literature on multivariate Laplace distributions, so additional research is needed. A starting point could be a generalization of the power exponential (*PE*) family of distributions (Gómez et al., 2007). Suppose the linear model includes *T* traits, and assume that residuals are distributed as **e**_*i*_~*PE*_*T*_(0, Σ_*T*×*T*_, β) where **Σ** is a positive-definite matrix and β>0 is a parameter relating to kurtosis and reflecting disparity from a multivariate normal distribution. A simple multiple-trait model can be represented as

(31) $$y_{i}=\gamma_{i}+g_{i}+e_{i};\;i\;=\;1,2,...,n,$$

where **y**_*i*_ and **g**_*i*_ are *T*×1 vectors of phenotypes and of genotypic values for individual *i*, respectively, and **γ**_*i*_ is a fixed *T*×1 vector. Conditionally on the genotypic values **g**_*i*_,one could assume that **y**_*i*_ has a *PE*_*T*_ ~(**y**_*i*_|**μ**_*i*_, Σ, β) distribution where **μ**_*i*_ = **γ**_*i*_+**g**_*i*_,and also that all **y**_*i*_ are conditionally independent. The sampling model would have density

(32) $$\begin{array}{l} p\left(y_{i}|\mu_{i},\Sigma ,\beta \right)=\frac{T\Gamma \left(\frac{T}{2}\right)}{\pi^{\frac{T}{2}}\Gamma \left(1+\frac{T}{2\beta }\right)2^{1+\frac{T}{2\beta }}{\left|\Sigma \right|}^{\frac{1}{2}}}\\ \;\exp\left\{-\frac{1}{2}{\left[{\left(y_{i}-\mu_{i}\right)}^{\prime }\Sigma^{-1}\left(y_{i}-\mu_{i}\right)\right]}^{\beta }\right\}. \end{array}$$

If β = 1 and *T* = 1 the density above becomes that of a univariate *N*(μ_*i*_, Σ) random variable as

(33) $$\begin{array}{l} p\left(y_{i}|\mu_{i},\Sigma ,\beta \right)=\frac{\Gamma \left(\frac{1}{2}\right)}{\pi^{\frac{1}{2}}\Gamma \left(\frac{3}{2}\right)\sqrt{2^{3}}{\left|\Sigma \right|}^{\frac{1}{2}}}\exp\left\{-\frac{1}{2\Sigma }{\left(y_{i}-\mu_{i}\right)}^{2}\right\}\\ \;=\frac{1}{\sqrt{2\pi \Sigma }}\exp\left\{-\frac{1}{2\Sigma }{\left(y_{i}-\mu_{i}\right)}^{2}\right\}, \end{array}$$

since $\Gamma \left(\frac{1}{2}\right)=\sqrt{\pi }$ and $\Gamma \left(\frac{3}{2}\right)=\frac{1}{2}\sqrt{\pi }.$ Now, using $\beta =\frac{1}{2}\;$and *T* = 1 in Equation (32) produces

$$\begin{array}{l} p\left(y_{i}|\mu_{i}\Sigma ,\beta \right)=\frac{\sqrt{\pi }}{\pi^{\frac{1}{2}}\Gamma \left(2\right)4{\left|\Sigma \right|}^{\frac{1}{2}}}\exp\left\{-\frac{1}{2\sqrt{\Sigma }}\left|y_{i}-\mu_{i}\right|\right\}\\ \;=\frac{1}{2m}\exp\left\{-\frac{1}{m}\left|y_{i}-\mu_{i}\right|\right\}, \end{array}$$

which is the density of a Laplace or DE distribution with mean μ_*i*_ and homogeneous (across observations) parameter $m=2\sqrt{\Sigma }.$ It follows that the *PE*_*T*_ ~(**μ**_*i*_, Σ, β) process provides a multivariate generalization of the *DE* distribution. However, developing a multi-trait LMAP is an item for future research and is beyond the scope of this paper. We also note that, if assigned to genotypic values or to markers in a whole-genome regression model, the distribution *PE*_*T*_ $\sim \left(g_{i}|0,\Sigma ,\beta =\frac{1}{2}\right)$ provides a prior for a multivariate Bayesian Lasso, which has not appeared in the quantitative genomics literature so far. Developing such a Bayesian model is an additional item for research although, given the notorious complexities of the single trait Bayesian Lasso, it is unlikely that such an approach (if and when developed) would lend itself to routine genetic evaluation of plants and animals.

Most traits in animal breeding have a heritability lower than 50% and, as already mentioned, many priors have been invented to better account for “genetic architecture” while retaining a naïve assumption for the error distribution. Also, there have been many attempts at enhancing predictive ability by including (not always with marked or fruitful results) non-additive genetic effects in the specification. Perhaps there is more opportunity for improving predictions by working on the residual part than on the genetic structure of a model. Super-dimensionality and a massive number of records can seldom cope with bad data, but robust models can mitigate damage stemming from concealed structure or by unknown sources of bias. Selection is not the only source of distortion in quantitative genetic analysis and the process is not always “ignorable” as almost always there is non-randomly missing data (Henderson et al., 1959; Rubin, 1976).

We underline that the data sets used in the present study were not “cherry picked,” i.e., there was no intentional bias or pre-selection of species or of traits that could be construed as favoring the thick-tailed distributions. Since every prediction exercise represents a different problem, it is impossible to anticipate how the proposed robust methodology will behave if applied to other traits and species. However, our results are encouraging and stimulate further investigation.

Our concluding statement, inspired by a famous Chinese book on military science (Sun Tzu, 6th century BC)^1^, and appropriate for the current “big data” environment, is

“*One can have an army with millions of soldiers, but if their weapon is just a fork, a smaller and better equipped rival can be more effective in battle."* [In other words, bigger is not necessarily better].

## Author contributions

DG conceived and conducted the analysis and drafted the manuscript. AC assisted with data analysis and manuscript. HN and C-CS assisted with the manuscript.

### Conflict of interest statement

The authors declare that the research was conducted in the absence of any commercial or financial relationships that could be construed as a potential conflict of interest.

## Footnotes

^1^Sun Tzu. 6th Century BC. *The Art of War*. Boulder, CO: Shambhala Publications.

## Supplementary material

Figure S1 Relationship between heritability and value of regularization parameters (Factor) in GBLUP (black open circles), TMAP4 (*t*—distribution with 4 degrees of freedom, red triangles) and LMAP (solid green circles). GBLUP: genomic BLUP with residual Gaussian distribution; TMAP4: maximum a posteriori with residual *t*—distribution on 4 degrees of freedom; LMAP: maximum a posteriori with double exponential residual distribution.

Figure S2 Quantile-quantile plot of milk yield in Italian Brown Swiss cows.

Figure S3 Quantile-quantile plot of flowering time, FRIGIDA gene expression and plant diameter in *Arabidopsis*.

Figure S4 Quantile-quantile plot of wheat yields 1–4.

Figure S5 Quantile-quantile plot of sums of pairs of wheat yields 1+2,…,3+4.

Figure S6 Quantile-quantile plot of sums of triplets of wheat yields 1+2+3,…,2+3+4.

Figure S7 Quantile-quantile plot of sums of tetraplet of wheat yields 1+2+3+4.
