# Supplementary figures and images for: Prediction of Complex Traits: Robust Alternatives to Best Linear Unbiased Prediction
Source: Front Genet. 2018 Jun 5;9:195. doi: 10.3389/fgene.2018.00195 (PMC6008589; doi:10.3389/fgene.2018.00195)

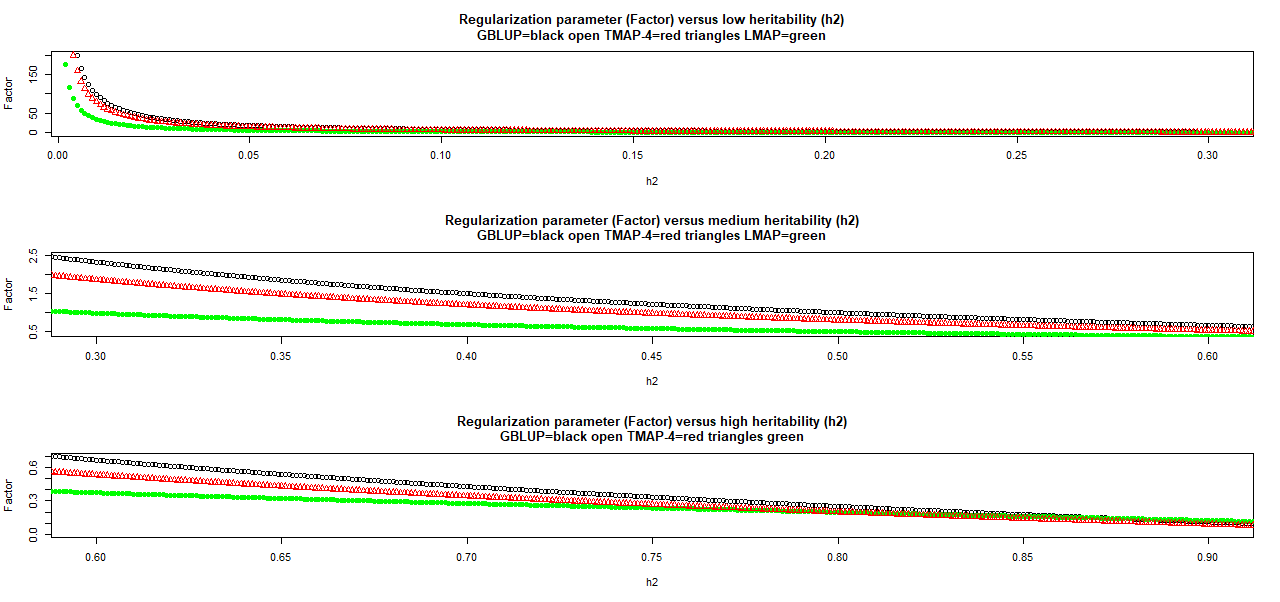

Supplement: Figure S1 — Relationship between heritability and value of regularization parameters (Factor) in GBLUP (black open circles), TMAP4 (t—distribution with 4 degrees of freedom, red triangles) and LMAP (solid green circles). GBLUP: genomic BLUP with residual Gaussian distribution; TMAP4: maximum a posteriori with residual t—distribution on 4 degrees of freedom; LMAP: maximum a posteriori with double exponential residual distribution. [file Image_1.TIFF]

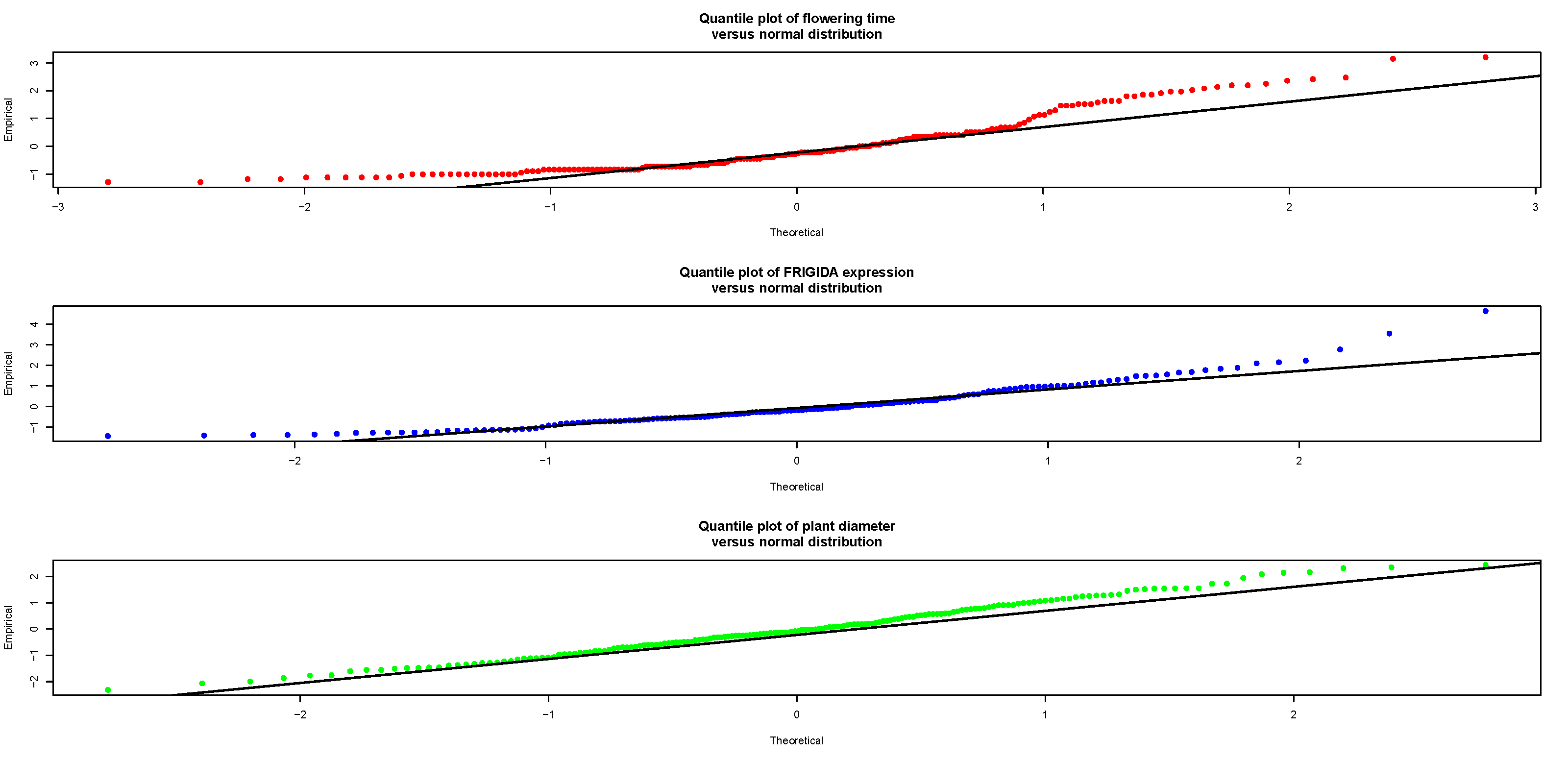

Supplement: Figure S3 — Quantile-quantile plot of flowering time, FRIGIDA gene expression and plant diameter in Arabidopsis. [file Image_3.TIFF]

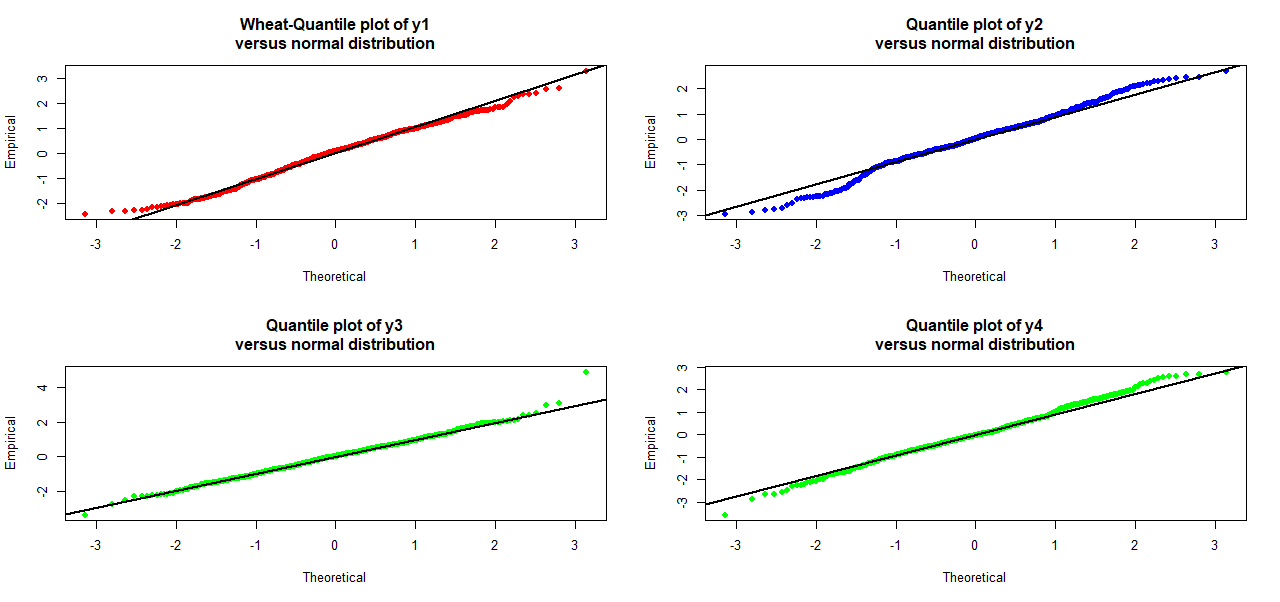

Supplement: Figure S4 — Quantile-quantile plot of wheat yields 1–4. [file Image_4.TIFF]

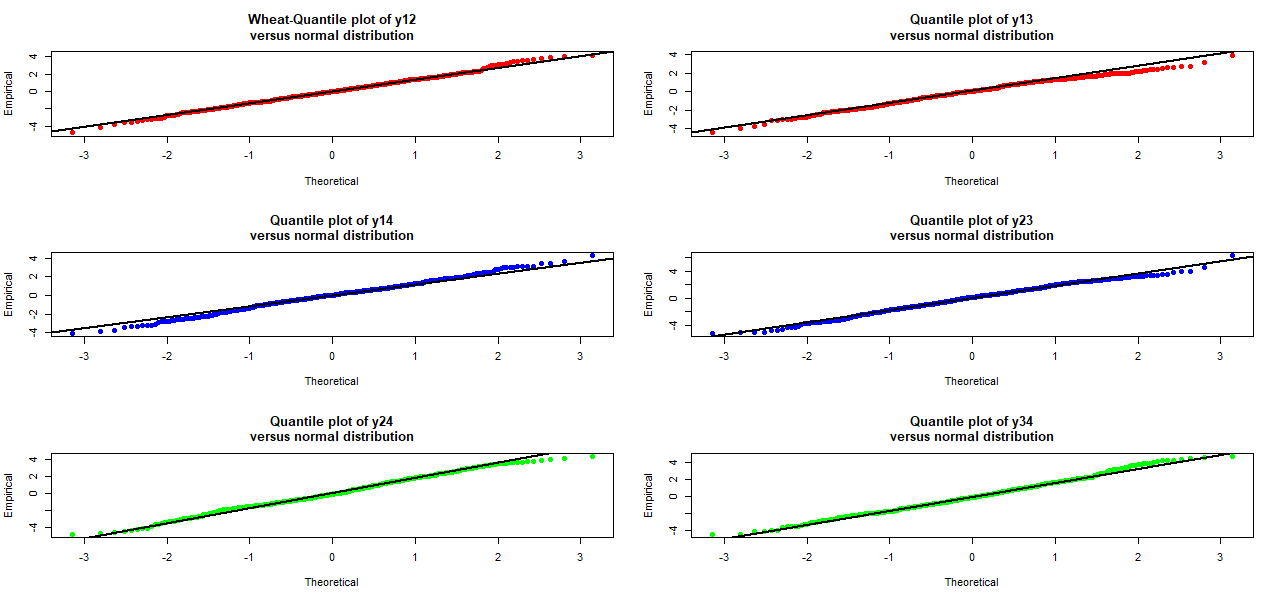

Supplement: Figure S5 — Quantile-quantile plot of sums of pairs of wheat yields 1+2,…,3+4. [file Image_5.TIFF]

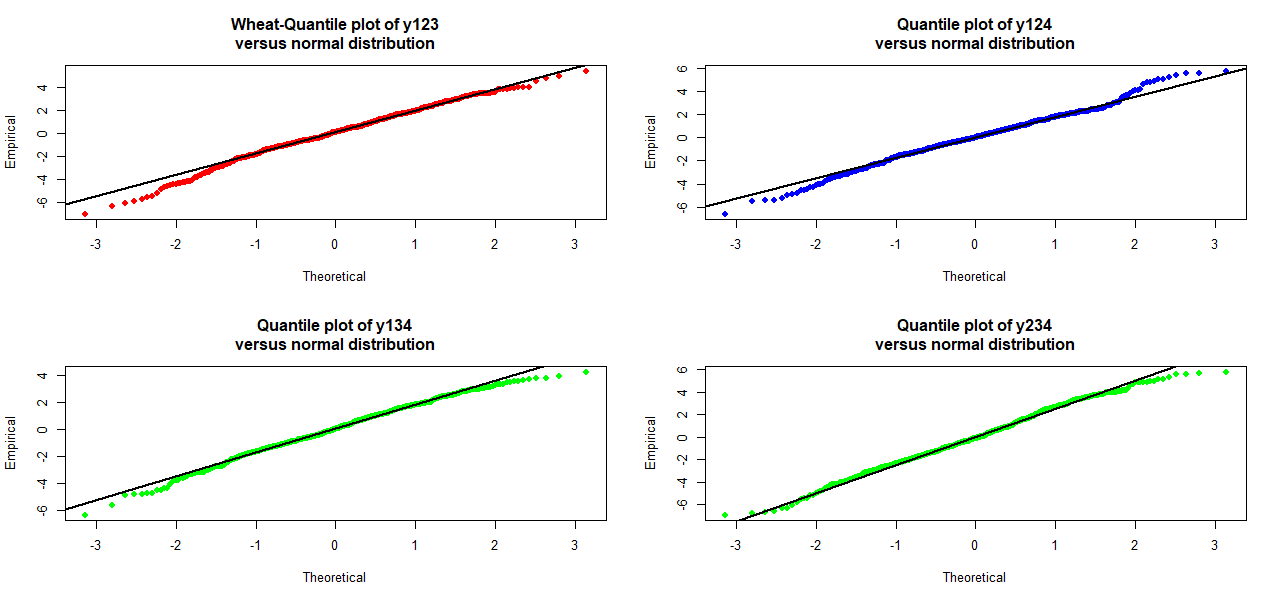

Supplement: Figure S6 — Quantile-quantile plot of sums of triplets of wheat yields 1+2+3,…,2+3+4. [file Image_6.TIFF]

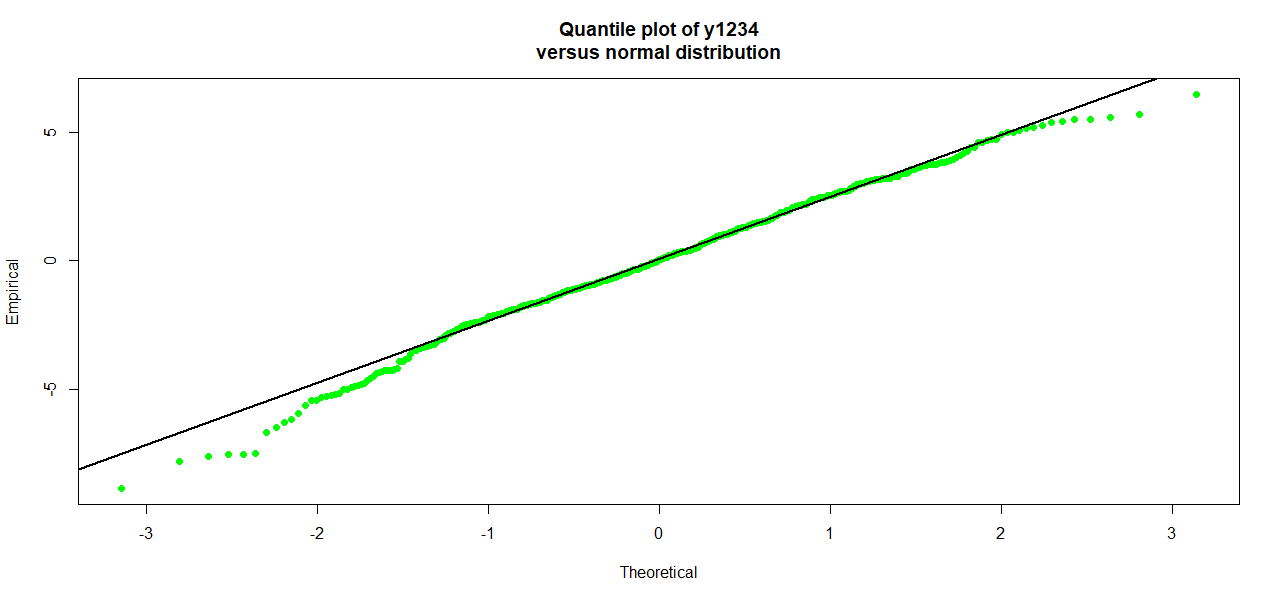

Supplement: Figure S7 — Quantile-quantile plot of sums of tetraplet of wheat yields 1+2+3+4. [file Image_7.TIFF]
