# Supplementary material for: Prediction of Complex Traits: Robust Alternatives to Best Linear Unbiased Prediction
Source: Front Genet. 2018 Jun 5;9:195. doi: 10.3389/fgene.2018.00195 (PMC6008589; doi:10.3389/fgene.2018.00195)
Supplement: Supplementary file 8 [file Data_Sheet_1.pdf]

Appendix to Prediction of complex traits: robust alternatives to best linear unbiased prediction

Daniel Gianola[a,b,c,d,e,1], Alessio Cecchinato[d], Hugo Naya[e], and Chris-Carolin Schön[c]

[a] Department of Animal Sciences, University of Wisconsin-Madison, USA;

[b] Department of Dairy Science, University of Wisconsin-Madison, USA;

[c] Department of Plant Sciences, Technical University of Munich, TUM School of Life Sciences, Germany;

[d] Department of Agronomy, Food Natural Resources, Animals and Environment University of Padova, Italy

[e] Institut Pasteur de Montevideo, Montevideo, Uruguay

[1] Corresponding author. Email: gianola@ansci.wisc.edu

May 28, 2018

1 APPENDIX A: Finding TMAP iteratively

1.1 First derivatives

The logarithm of the conditional posterior density given in Equation 6, apart from an additive constant, is

$$L(\mathbf{g},\boldsymbol{\alpha}) = -\frac{(\nu+1)}{2}\sum_{i=1}^{n}\log\left[1+\frac{n_i}{\tau_e^2\nu}\left(y_i-\mathbf{w}_i'\boldsymbol{\alpha}-\mathbf{z}_i'\mathbf{g}\right)^2\right]-\frac{1}{2\sigma_g^2}\mathbf{g}'\mathbf{K}^{-1}\mathbf{g}. \tag{1}$$

The gradient with respect to $\boldsymbol{\alpha}$ is

$$\frac{\partial L(\mathbf{g},\boldsymbol{\alpha})}{\partial\boldsymbol{\alpha}} = (\nu+1)\sum_{i=1}^{n}\frac{\frac{n_i}{\tau_e^2\nu}\mathbf{w}_i\left(y_i-\mathbf{w}_i'\boldsymbol{\alpha}-\mathbf{z}_i'\mathbf{g}\right)}{\left[1+\frac{n_i}{\tau_e^2\nu}\left(y_i-\mathbf{w}_i'\boldsymbol{\alpha}-\mathbf{z}_i'\mathbf{g}\right)^2\right]}, \tag{2}$$

whereas the $\mathbf{g}-$gradient is

$$\frac{\partial L(\mathbf{g},\boldsymbol{\alpha})}{\partial \mathbf{g}} = (\nu+1)\sum_{i=1}^{n}\frac{\frac{n_i}{\tau_e^2\nu}\mathbf{z}_i\left(y_i-\mathbf{w}_i'\boldsymbol{\alpha}-\mathbf{z}_i'\mathbf{g}\right)}{\left[1+\frac{n_i}{\tau_e^2\nu}\left(y_i-\mathbf{w}_i'\boldsymbol{\alpha}-\mathbf{z}_i'\mathbf{g}\right)^2\right]}-\frac{1}{\sigma_g^2}\mathbf{K}^{-1}\mathbf{g}. \tag{3}$$

Note that $\mathbf{W}'\mathbf{W}=\sum_{i=1}^{n}\mathbf{w}_i\mathbf{w}_i'$, $\mathbf{Z}'\mathbf{Z}=\sum_{i=1}^{n}\mathbf{z}_i\mathbf{z}_i'$, and let

$$d_i = \frac{n_i}{\left[1+\frac{\left(y_i-\mathbf{w}_i'\boldsymbol{\alpha}-\mathbf{z}_i'\mathbf{g}\right)^2}{\frac{\tau_e^2}{n_i}\nu}\right]}, \tag{4}$$

be a positive "weight" assigned to observation i, with $\mathbf{D}=diag\{d_i\}$ being an $n\times n$ diagonal matrix of such weights. Using the preceding definitions, the gradient vectors become

$$\begin{aligned}\frac{\partial L(\mathbf{g},\boldsymbol{\alpha})}{\partial \boldsymbol{\alpha}} &= \frac{(\nu+1)}{\tau_e^2\nu}\sum_{i=1}^{n}d_i\mathbf{w}_i\left(y_i-\mathbf{w}_i'\boldsymbol{\alpha}-\mathbf{z}_i'\mathbf{g}\right)\\ &= \frac{(\nu+1)}{\tau_e^2\nu}\left(\mathbf{W}'\mathbf{D}\mathbf{y}-\mathbf{W}'\mathbf{D}\mathbf{W}\boldsymbol{\alpha}-\mathbf{W}'\mathbf{D}\mathbf{Z}\mathbf{g}\right) \end{aligned} \tag{5}$$

and

$$\begin{aligned}\frac{\partial L(\mathbf{g},\boldsymbol{\alpha})}{\partial \mathbf{g}} &= \frac{(\nu+1)}{\tau_e^2\nu}\sum_{i=1}^{n}d_i\mathbf{z}_i\left(y_i-\mathbf{w}_i'\boldsymbol{\alpha}-\mathbf{z}_i'\mathbf{g}\right)-\frac{1}{\sigma_g^2}\mathbf{K}^{-1}\mathbf{g}\\ &= \frac{(\nu+1)}{\tau_e^2\nu}\left(\mathbf{Z}'\mathbf{D}\mathbf{y}-\mathbf{Z}'\mathbf{D}\mathbf{W}\boldsymbol{\alpha}-\mathbf{Z}'\mathbf{D}\mathbf{Z}\mathbf{g}\right)-\frac{1}{\sigma_g^2}\mathbf{K}^{-1}\mathbf{g}. \end{aligned} \tag{6}$$

Set now the differentials to zero, to meet the first-order condition for a maximum, and rearrange expressions, producing equations

$$\mathbf{W}'\mathbf{D}\mathbf{W}\boldsymbol{\alpha}+\mathbf{W}'\mathbf{D}\mathbf{Z}\mathbf{g} = \mathbf{W}'\mathbf{D}\mathbf{y} \tag{7}$$

$$\mathbf{Z}'\mathbf{D}\mathbf{W}\boldsymbol{\alpha}+\left(\mathbf{Z}'\mathbf{D}\mathbf{Z}+\frac{\tau_e^2\nu}{\sigma_g^2(\nu+1)}\mathbf{K}^{-1}\right)\mathbf{g} = \mathbf{Z}'\mathbf{D}\mathbf{y}. \tag{8}$$

These equations are not explicit because the d_i $(i=1,2,...,n)$ depend on $\boldsymbol{\alpha}$ and $\mathbf{g}$. A functional

iteration can be established, with matrix form

$$\begin{bmatrix} \mathbf{W}'\mathbf{D}^{[t]}\mathbf{W} & \mathbf{W}'\mathbf{D}^{[t]}\mathbf{Z} \\ \mathbf{Z}'\mathbf{D}^{[t]}\mathbf{W} & \mathbf{Z}'\mathbf{D}^{[t]}\mathbf{Z}+\dfrac{\lambda'\nu}{(\nu+1)}\mathbf{K}^{-1} \end{bmatrix} \begin{bmatrix} \boldsymbol{\alpha}^{[t+1]} \\ \mathbf{g}^{[t+1]} \end{bmatrix} = \begin{bmatrix} \mathbf{W}'\mathbf{D}^{[t]}\mathbf{y} \\ \mathbf{Z}'\mathbf{D}^{[t]}\mathbf{y} \end{bmatrix}, \tag{9}$$

where $\lambda' = \dfrac{\tau_e^2}{\sigma_g^2}$. Starting d_i values (iteration 0) can be computed by assuming some value of heritability or of λ', fixing ν at some point and setting $\boldsymbol{\alpha}$ and $\mathbf{g}$ to the values obtained with the Gaussian model, say. As indicated in the main body of the paper, a predictive approach can be used for calibration of λ' and ν, acting as regularization parameters.

1.2 Second derivatives and Newton-Raphson algorithm

The NR algorithm iterates with

$$\begin{bmatrix} -\dfrac{\partial^2 L(\mathbf{g},\boldsymbol{\alpha})}{\partial\boldsymbol{\alpha}\partial\boldsymbol{\alpha}'} & -\dfrac{\partial^2 L(\mathbf{g},\boldsymbol{\alpha})}{\partial\boldsymbol{\alpha}\partial\mathbf{g}'} \\ -\dfrac{\partial^2 L(\mathbf{g},\boldsymbol{\alpha})}{\partial\mathbf{g}\partial\boldsymbol{\alpha}'} & -\dfrac{\partial^2 L(\mathbf{g},\boldsymbol{\alpha})}{\partial\mathbf{g}\partial\mathbf{g}'} \end{bmatrix}^{[t]} \begin{bmatrix} \boldsymbol{\alpha}^{[t+1]} \\ \mathbf{g}^{[t+1]} \end{bmatrix} = \begin{bmatrix} -\dfrac{\partial^2 L(\mathbf{g},\boldsymbol{\alpha})}{\partial\boldsymbol{\alpha}\partial\boldsymbol{\alpha}'} & -\dfrac{\partial^2 L(\mathbf{g},\boldsymbol{\alpha})}{\partial\boldsymbol{\alpha}\partial\mathbf{g}'} \\ -\dfrac{\partial^2 L(\mathbf{g},\boldsymbol{\alpha})}{\partial\mathbf{g}\partial\boldsymbol{\alpha}'} & -\dfrac{\partial^2 L(\mathbf{g},\boldsymbol{\alpha})}{\partial\mathbf{g}\partial\mathbf{g}'} \end{bmatrix}^{[t]} \begin{bmatrix} \boldsymbol{\alpha}^{[t]} \\ \mathbf{g}^{[t]} \end{bmatrix} + \begin{bmatrix} \dfrac{\partial L_{DE}}{\partial\boldsymbol{\alpha}} \\ \dfrac{\partial L_{DE}}{\partial\mathbf{g}} \end{bmatrix}^{[t]}. \tag{10}$$

Using results from expressions (2), (3) and (4)

$$\begin{aligned} \frac{\partial^2 L(\mathbf{g},\boldsymbol{\alpha})}{\partial\boldsymbol{\alpha}\partial\boldsymbol{\alpha}'} &= \frac{(\nu+1)}{\tau_e^2\nu}\sum_{i=1}^{n}\left\{d_i\left[\frac{\partial}{\partial\boldsymbol{\alpha}'}\mathbf{w}_i(y_i-\mathbf{w}_i'\boldsymbol{\alpha}-\mathbf{z}_i'\mathbf{g})\right]\right. \\ &\quad \left.+\left(\frac{\partial}{\partial\boldsymbol{\alpha}'}d_i\right)\mathbf{w}_i(y_i-\mathbf{w}_i'\boldsymbol{\alpha}-\mathbf{z}_i'\mathbf{g})\right\}. \end{aligned} \tag{11}$$

Here

$$\frac{\partial}{\partial\boldsymbol{\alpha}'}d_i = \frac{\partial}{\partial\boldsymbol{\alpha}'}\left\{\frac{n_i}{\left[1+\dfrac{(y_i-\mathbf{w}_i'\boldsymbol{\alpha}-\mathbf{z}_i'\mathbf{g})^2}{\dfrac{\tau_e^2}{n_i}\nu}\right]}\right\} = \frac{2n_i\dfrac{(y_i-\mathbf{w}_i'\boldsymbol{\alpha}-\mathbf{z}_i'\mathbf{g})}{\dfrac{\tau_e^2}{n_i}\nu}}{\left[1+\dfrac{(y_i-\mathbf{w}_i'\boldsymbol{\alpha}-\mathbf{z}_i'\mathbf{g})^2}{\dfrac{\tau_e^2}{n_i}\nu}\right]^2}\mathbf{w}_i'. \tag{12}$$

Using (12) in (11)

$$\begin{aligned}
\frac{\partial^2 L(\mathbf{g},\boldsymbol{\alpha})}{\partial\boldsymbol{\alpha}\partial\boldsymbol{\alpha}'} &= -\frac{(\nu+1)}{\tau_e^2\nu}\mathbf{W}'\mathbf{D}^{[t]}\mathbf{W}+\frac{(\nu+1)}{\tau_e^2\nu}\sum_{i=1}^{n}\mathbf{w}_i\mathbf{w}_i'\frac{2n_i\dfrac{(y_i-\mathbf{w}_i'\boldsymbol{\alpha}-\mathbf{z}_i'\mathbf{g})^2}{\frac{\tau_e^2}{n_i}\nu}}{\left[1+\dfrac{(y_i-\mathbf{w}_i'\boldsymbol{\alpha}-\mathbf{z}_i'\mathbf{g})^2}{\frac{\tau_e^2}{n_i}\nu}\right]^2}\\
&= -\frac{(\nu+1)}{\tau_e^2\nu}\mathbf{W}'\mathbf{D}^{[t]}\mathbf{W}+\frac{2(\nu+1)}{\tau_e^2\nu}\sum_{i=1}^{n}\mathbf{w}_i\mathbf{w}_i'd_i\frac{(y_i-\mathbf{w}_i'\boldsymbol{\alpha}-\mathbf{z}_i'\mathbf{g})^2}{\frac{\tau_e^2}{n_i}\nu\left[1+\dfrac{(y_i-\mathbf{w}_i'\boldsymbol{\alpha}-\mathbf{z}_i'\mathbf{g})^2}{\frac{\tau_e^2}{n_i}\nu}\right]}\\
&= -\frac{(\nu+1)}{\tau_e^2\nu}\mathbf{W}'\mathbf{D}^{[t]}\mathbf{W}+\frac{2(\nu+1)}{\tau_e^2\nu}\sum_{i=1}^{n}\mathbf{w}_i\mathbf{w}_i'd_i\frac{(y_i-\mathbf{w}_i'\boldsymbol{\alpha}-\mathbf{z}_i'\mathbf{g})^2}{\frac{\tau_e^2}{n_i}\nu\frac{n_i}{d_i}}\\
&= -\frac{(\nu+1)}{\tau_e^2\nu}\mathbf{W}'\mathbf{D}^{[t]}\mathbf{W}+\frac{2(\nu+1)}{\tau_e^2\nu}\mathbf{W}'\mathbf{D}^{[t]}\mathbf{S}^{[t]}\mathbf{D}^{[t]}\mathbf{W}, \qquad (13)
\end{aligned}$$

where $\mathbf{S}^{[t]} = Diag\{s_i\}$ is an $n\times n$ diagonal matrix and $s_i = \frac{(y_i-\mathbf{w}_i'\boldsymbol{\alpha}-\mathbf{z}_i'\mathbf{g})^2}{\tau_e^2\nu}$. Hence, for $\mathbf{Q}^{[t]} = \mathbf{D}^{[t]} - 2\mathbf{D}^{[t]}\mathbf{S}^{[t]}\mathbf{D}^{[t]}$

$$\begin{aligned}
\frac{\partial^2 L(\mathbf{g},\boldsymbol{\alpha})}{\partial\boldsymbol{\alpha}\partial\boldsymbol{\alpha}'} &= -\frac{(\nu+1)}{\tau_e^2\nu}\mathbf{W}'\mathbf{Q}^{[t]}\mathbf{W}, \qquad (14)\\
\frac{\partial^2 L(\mathbf{g},\boldsymbol{\alpha})}{\partial\boldsymbol{\alpha}\partial\mathbf{g}'} &= -\frac{(\nu+1)}{\tau_e^2\nu}\mathbf{W}'\mathbf{Q}^{[t]}\mathbf{Z}, \qquad (15)\\
\frac{\partial^2 L(\mathbf{g},\boldsymbol{\alpha})}{\partial\mathbf{g}\partial\mathbf{g}'} &= -\frac{(\nu+1)}{\tau_e^2\nu}\mathbf{Z}'\mathbf{Q}^{[t]}\mathbf{Z}-\frac{1}{\sigma_g^2}\mathbf{K}^{-1}. \qquad (16)
\end{aligned}$$

Employing the first and second differentials presented above, NR becomes

$$
\begin{bmatrix} \frac{(\nu+1)}{\tau_e^2\nu}\mathbf{W}'\mathbf{Q}^{[t]}\mathbf{W} & \frac{(\nu+1)}{\tau_e^2\nu}\mathbf{W}'\mathbf{Q}^{[t]}\mathbf{Z} \\ \frac{(\nu+1)}{\tau_e^2\nu}\mathbf{Z}'\mathbf{Q}^{[t]}\mathbf{W} & \frac{(\nu+1)}{\tau_e^2\nu}\mathbf{Z}'\mathbf{Q}^{[t]}\mathbf{Z}+\frac{1}{\sigma_g^2}\mathbf{K}^{-1} \end{bmatrix} \begin{bmatrix} \boldsymbol{\alpha}^{[t+1]} \\ \mathbf{g}^{[t+1]} \end{bmatrix}
$$

$$
= \begin{bmatrix} \frac{(\nu+1)}{\tau_e^2\nu}\mathbf{W}'\mathbf{Q}^{[t]}\mathbf{W} & \frac{(\nu+1)}{\tau_e^2\nu}\mathbf{W}'\mathbf{Q}^{[t]}\mathbf{Z} \\ \frac{(\nu+1)}{\tau_e^2\nu}\mathbf{Z}'\mathbf{Q}^{[t]}\mathbf{W} & \frac{(\nu+1)}{\tau_e^2\nu}\mathbf{Z}'\mathbf{Q}^{[t]}\mathbf{Z}+\frac{1}{\sigma_g^2}\mathbf{K}^{-1} \end{bmatrix} \begin{bmatrix} \boldsymbol{\alpha}^{[t]} \\ \mathbf{g}^{[t]} \end{bmatrix}
+ \begin{bmatrix} \frac{(\nu+1)}{\tau_e^2\nu}\mathbf{W}'\mathbf{D}^{[t]}\left(\mathbf{y}-\mathbf{W}\boldsymbol{\alpha}^{[t]}-\mathbf{Z}\mathbf{g}^{[t]}\right) \\ \frac{(\nu+1)}{\tau_e^2\nu}\mathbf{Z}'\mathbf{D}^{[t]}\left(\mathbf{y}-\mathbf{W}\boldsymbol{\alpha}^{[t]}-\mathbf{Z}\mathbf{g}^{[t]}\right)-\frac{1}{\sigma_g^2}\mathbf{K}^{-1}\mathbf{g}^{[t]} \end{bmatrix}. \tag{17}
$$

One can obtain an alternative representation by multiplying both sides by $\frac{\tau_e^2\nu}{(\nu+1)}$, producing

$$
\begin{bmatrix} \mathbf{W}'\mathbf{Q}^{[t]}\mathbf{W} & \mathbf{W}'\mathbf{Q}^{[t]}\mathbf{Z} \\ \mathbf{Z}'\mathbf{Q}^{[t]}\mathbf{W} & \mathbf{Z}'\mathbf{Q}^{[t]}\mathbf{Z}+\lambda\frac{\nu}{(\nu+1)}\mathbf{K}^{-1} \end{bmatrix} \begin{bmatrix} \boldsymbol{\alpha}^{[t+1]} \\ \mathbf{g}^{[t+1]} \end{bmatrix}
$$

$$
= \begin{bmatrix} \mathbf{W}'\mathbf{Q}^{[t]}\mathbf{W} & \mathbf{W}'\mathbf{Q}^{[t]}\mathbf{Z} \\ \mathbf{Z}'\mathbf{Q}^{[t]}\mathbf{W} & \mathbf{Z}'\mathbf{Q}^{[t]}\mathbf{Z}+\lambda'\frac{\nu}{(\nu+1)}\mathbf{K}^{-1} \end{bmatrix} \begin{bmatrix} \boldsymbol{\alpha}^{[t]} \\ \mathbf{g}^{[t]} \end{bmatrix}
+ \begin{bmatrix} \mathbf{W}'\mathbf{D}^{[t]}\left(\mathbf{y}-\mathbf{W}\boldsymbol{\alpha}^{[t]}-\mathbf{Z}\mathbf{g}^{[t]}\right) \\ \mathbf{Z}'\mathbf{D}^{[t]}\left(\mathbf{y}-\mathbf{W}\boldsymbol{\alpha}^{[t]}-\mathbf{Z}\mathbf{g}^{[t]}\right)-\lambda'\frac{\nu}{(\nu+1)}\mathbf{K}^{-1}\mathbf{g}^{[t]} \end{bmatrix}. \tag{18}
$$

The right-hand side vector in (18) can be written as

$$
\begin{bmatrix} \mathbf{W}'\mathbf{Q}^{[t]}\mathbf{W}\boldsymbol{\alpha}^{[t]}+\mathbf{W}'\mathbf{Q}^{[t]}\mathbf{Z}\mathbf{g}^{[t]}+\mathbf{W}'\mathbf{D}^{[t]}\left(\mathbf{y}-\mathbf{W}\boldsymbol{\alpha}^{[t]}-\mathbf{Z}\mathbf{g}^{[t]}\right) \\ \mathbf{Z}'\mathbf{Q}^{[t]}\mathbf{W}\boldsymbol{\alpha}^{[t]}+\mathbf{Z}'\mathbf{Q}^{[t]}\mathbf{Z}\mathbf{g}^{[t]}+\mathbf{Z}'\mathbf{D}^{[t]}\left(\mathbf{y}-\mathbf{W}\boldsymbol{\alpha}^{[t]}-\mathbf{Z}\mathbf{g}^{[t]}\right) \end{bmatrix}
$$

$$
= \begin{bmatrix} \mathbf{W}'\mathbf{Q}^{[t]}\left[\mathbf{W}\boldsymbol{\alpha}^{[t]}+\mathbf{Z}\mathbf{g}^{[t]}+\mathbf{Q}^{[t]-1}\mathbf{D}^{[t]}\left(\mathbf{y}-\mathbf{W}\boldsymbol{\alpha}^{[t]}-\mathbf{Z}\mathbf{g}^{[t]}\right)\right] \\ \mathbf{Z}'\mathbf{Q}^{[t]}\left[\mathbf{W}\boldsymbol{\alpha}^{[t]}+\mathbf{Z}\mathbf{g}^{[t]}+\mathbf{Q}^{[t]-1}\mathbf{D}^{[t]}\left(\mathbf{y}-\mathbf{W}\boldsymbol{\alpha}^{[t]}-\mathbf{Z}\mathbf{g}^{[t]}\right)\right] \end{bmatrix} = \begin{bmatrix} \mathbf{W}'\mathbf{Q}^{[t]}\mathbf{y}^{[t]} \\ \mathbf{Z}'\mathbf{Q}^{[t]}\mathbf{y}^{[t]} \end{bmatrix}, \tag{19}
$$

where $\mathbf{y}^{[t]} = \mathbf{W}\boldsymbol{\alpha}^{[t]}+\mathbf{Z}\mathbf{g}^{[t]}+\mathbf{Q}^{[t]-1}\mathbf{D}^{[t]}\left(\mathbf{y}-\mathbf{W}\boldsymbol{\alpha}^{[t]}-\mathbf{Z}\mathbf{g}^{[t]}\right)$ is a "working phenotype". Finally, employing (19) in (18), the Newton-Raphson algorithm becomes the following iterative re-weighted set of mixed model equations

$$
\begin{bmatrix} \mathbf{W}'\mathbf{Q}^{[t]}\mathbf{W} & \mathbf{W}'\mathbf{Q}^{[t]}\mathbf{Z} \\ \mathbf{Z}'\mathbf{Q}^{[t]}\mathbf{W} & \mathbf{Z}'\mathbf{Q}^{[t]}\mathbf{Z}+\lambda'\frac{\nu}{(\nu+1)}\mathbf{K}^{-1} \end{bmatrix} \begin{bmatrix} \boldsymbol{\alpha}^{[t+1]} \\ \mathbf{g}^{[t+1]} \end{bmatrix} = \begin{bmatrix} \mathbf{W}'\mathbf{Q}^{[t]}\mathbf{y}^{[t]} \\ \mathbf{Z}'\mathbf{Q}^{[t]}\mathbf{y}^{[t]} \end{bmatrix}, \tag{20}
$$

implying that the equations can be solved via repeated application of any fast solver of mixed model equations.

2 APPENDIX B: Finding LMAP iteratively

Consider Equation 17

$$\begin{aligned} L_{DE} &= \log p\left(\mathbf{g}, \boldsymbol{\alpha}|\sigma_e^2, \sigma_g^2, \mathbf{y}\right) \\ &= C - \frac{1}{\sqrt{\frac{\sigma_e^2}{2}}}\sum_{i=1}^{n}\sqrt{n_i}\left|y_i - \mu_i\right| - \frac{1}{2\sigma_g^2}\mathbf{g}'\mathbf{K}^{-1}\mathbf{g}, \end{aligned} \tag{21}$$

and recall that $\mu_i = \mathbf{w}_i'\boldsymbol{\alpha} + \mathbf{z}_i'\mathbf{g}$. The sum involving absolute values can be put in form

$$\sum_{i=1}^{n}\sqrt{n_i}\left|y_i - \mu_i\right| = \sum_{i=1}^{n}\sqrt{n_i}\frac{\left(y_i - \mu_i\right)^2}{\left|y_i - \mu_i\right|} = \left(\mathbf{y} - \mathbf{W}\boldsymbol{\alpha} - \mathbf{Z}\mathbf{g}\right)'\mathbf{M}\left(\mathbf{y} - \mathbf{W}\boldsymbol{\alpha} - \mathbf{Z}\mathbf{g}\right), \tag{22}$$

where $\mathbf{M} = Diag\left\{m_i\right\}$ is a diagonal $m \times m$ matrix with typical element $\sqrt{n_i}\left|y_i - \mu_i\right|^{-1}$, which exists if $\mu_i \neq y_i$; note that $\frac{\left(y_i - \mu_i\right)}{\left|y_i - \mu_i\right|} = sign\left(y_i - \mu_i\right)$. If the $\mu_i's$ in $\mathbf{M}$ are replaced by some numbers and regarded as constants, producing matrix $\mathbf{M}^{[0]}$, say, then in the neighborhood of $\mathbf{M}^{[0]}$

$$\begin{aligned} \frac{\partial L_{DE}}{\partial \boldsymbol{\alpha}} &\approx -\frac{1}{\sqrt{\frac{\sigma_e^2}{2}}}\frac{\partial}{\partial \boldsymbol{\alpha}}\left(\mathbf{y} - \mathbf{W}\boldsymbol{\alpha} - \mathbf{Z}\mathbf{g}\right)'\mathbf{M}^{[0]}\left(\mathbf{y} - \mathbf{W}\boldsymbol{\alpha} - \mathbf{Z}\mathbf{g}\right) \\ &= \frac{2}{\sqrt{\frac{\sigma_e^2}{2}}}\mathbf{W}'\mathbf{M}^{[0]}\left(\mathbf{y} - \mathbf{W}\boldsymbol{\alpha} - \mathbf{Z}\mathbf{g}\right). \end{aligned} \tag{23}$$

Similarly

$$\frac{\partial L_{DE}}{\partial \mathbf{g}} = \frac{2}{\sqrt{\frac{\sigma_e^2}{2}}}\mathbf{Z}'\mathbf{M}^{[0]}\left(\mathbf{y} - \mathbf{W}\boldsymbol{\alpha} - \mathbf{Z}\mathbf{g}\right) - \frac{1}{\sigma_g^2}\mathbf{K}^{-1}\mathbf{g}. \tag{24}$$

Setting (23) and (24) simultaneously to $\mathbf{0}$, one obtains an implicit system of equations that can be solved repeatedly via iteration with matrix expression

$$\begin{bmatrix} \mathbf{W}'\mathbf{M}^{[t]}\mathbf{W} & \mathbf{W}'\mathbf{M}^{[t]}\mathbf{Z} \\ \mathbf{Z}'\mathbf{M}^{[t]}\mathbf{W} & \mathbf{Z}'\mathbf{M}^{[t]}\mathbf{Z} + \omega\mathbf{K}^{-1} \end{bmatrix}\begin{bmatrix} \boldsymbol{\alpha}^{[t+1]} \\ \mathbf{g}^{[t+1]} \end{bmatrix} = \begin{bmatrix} \mathbf{W}'\mathbf{M}^{[t]}\mathbf{y} \\ \mathbf{Z}'\mathbf{M}^{[t]}\mathbf{y} \end{bmatrix}, \tag{25}$$

where $\omega = \frac{\sqrt{\frac{\sigma_e^2}{2}}}{2\sigma_g^2}$ is a regularization parameter.

As in the case of TMAP, an algorithm based on second-derivatives (e.g., NR) might reach a global mode faster provided starting values are "good enough". The second differentials needed (again, treating the $\mu's$ "inside" of $\mathbf{M}$ as constants) are

$$\frac{\partial^2 L_{DE}}{\partial\boldsymbol{\alpha}\partial\boldsymbol{\alpha}'} \approx \frac{\partial}{\partial\boldsymbol{\alpha}'}\left[\frac{2}{\sqrt{\frac{\sigma_e^2}{2}}}\mathbf{W}'\mathbf{M}^{[0]}\left(\mathbf{y}-\mathbf{W}\boldsymbol{\alpha}-\mathbf{Z}\mathbf{g}\right)\right] = -\frac{2}{\sqrt{\frac{\sigma_e^2}{2}}}\mathbf{W}'\mathbf{M}^{[0]}\mathbf{W}, \tag{26}$$

$$\frac{\partial^2 L_{DE}}{\partial\boldsymbol{\alpha}\partial\mathbf{g}'} \approx -\frac{2}{\sqrt{\frac{\sigma_e^2}{2}}}\mathbf{W}'\mathbf{M}^{[0]}\mathbf{Z}, \tag{27}$$

$$\frac{\partial^2 L_{DE}}{\partial\mathbf{g}\partial\boldsymbol{\alpha}'} \approx -\frac{2}{\sqrt{\frac{\sigma_e^2}{2}}}\mathbf{Z}'\mathbf{M}^{[0]}\mathbf{W}, \tag{28}$$

$$\frac{\partial^2 L_{DE}}{\partial\mathbf{g}\partial\mathbf{g}'} \approx -\frac{2}{\sqrt{\frac{\sigma_e^2}{2}}}\mathbf{Z}'\mathbf{M}^{[0]}\mathbf{Z}-\frac{1}{\sigma_g^2}\mathbf{K}^{-1}. \tag{29}$$

NR here takes the form

$$\begin{bmatrix} \frac{2}{\sqrt{\frac{\sigma_e^2}{2}}}\mathbf{W}'\mathbf{M}^{[t]}\mathbf{W} & \frac{2}{\sqrt{\frac{\sigma_e^2}{2}}}\mathbf{W}'\mathbf{M}^{[t]}\mathbf{Z} \\ \frac{2}{\sqrt{\frac{\sigma_e^2}{2}}}\mathbf{Z}'\mathbf{M}^{[t]}\mathbf{W} & \frac{2}{\sqrt{\frac{\sigma_e^2}{2}}}\mathbf{Z}'\mathbf{M}^{[t]}\mathbf{Z}+\frac{1}{\sigma_g^2}\mathbf{K}^{-1} \end{bmatrix}\begin{bmatrix}\boldsymbol{\alpha}^{[t+1]} \\ \mathbf{g}^{[t+1]}\end{bmatrix}$$
$$= \begin{bmatrix} \frac{2}{\sqrt{\frac{\sigma_e^2}{2}}}\mathbf{W}'\mathbf{M}^{[t]}\mathbf{W} & \frac{2}{\sqrt{\frac{\sigma_e^2}{2}}}\mathbf{W}'\mathbf{M}^{[t]}\mathbf{Z} \\ \frac{2}{\sqrt{\frac{\sigma_e^2}{2}}}\mathbf{Z}'\mathbf{M}^{[t]}\mathbf{W} & \frac{2}{\sqrt{\frac{\sigma_e^2}{2}}}\mathbf{Z}'\mathbf{M}^{[t]}\mathbf{Z}+\frac{1}{\sigma_g^2}\mathbf{K}^{-1} \end{bmatrix}\begin{bmatrix}\boldsymbol{\alpha}^{[t]} \\ \mathbf{g}^{[t]}\end{bmatrix}$$
$$+\begin{bmatrix} \frac{2}{\sqrt{\frac{\sigma_e^2}{2}}}\mathbf{W}'\mathbf{M}^{[t]}\left(\mathbf{y}-\mathbf{W}\boldsymbol{\alpha}^{[t]}-\mathbf{Z}\mathbf{g}^{[t]}\right) \\ \frac{2}{\sqrt{\frac{\sigma_e^2}{2}}}\mathbf{Z}'\mathbf{M}^{[t]}\left(\mathbf{y}-\mathbf{W}\boldsymbol{\alpha}^{[t]}-\mathbf{Z}\mathbf{g}^{[t]}\right)-\frac{1}{\sigma_g^2}\mathbf{K}^{-1}\mathbf{g}^{[t]} \end{bmatrix}, \tag{30}$$

After cancelling terms,

$$\begin{bmatrix} \mathbf{W}'\mathbf{M}^{[t]}\mathbf{W} & \mathbf{W}'\mathbf{M}^{[t]}\mathbf{Z} \\ \mathbf{Z}'\mathbf{M}^{[t]}\mathbf{W} & \mathbf{Z}'\mathbf{M}^{[t]}\mathbf{Z}+\omega\mathbf{K}^{-1} \end{bmatrix} \begin{bmatrix} \boldsymbol{\alpha}^{[t+1]} \\ \mathbf{g}^{[t+1]} \end{bmatrix} = \begin{bmatrix} \mathbf{W}'\mathbf{M}^{[t]}\mathbf{y} \\ \mathbf{Z}'\mathbf{M}^{[t]}\mathbf{W}\mathbf{y} \end{bmatrix}. \tag{31}$$

It follows that, when using the approximation indicated above in the process of differentiation, the NR algorithm and the functional iteration suggested earlier in this Appendix represent the same numerical procedure. However, the second derivatives from NR can be used to approximate the covariance matrix of the conditional posterior distribution ($t = \infty$ means "after convergence to maximum"):

$$\begin{aligned} \widehat{Var}\left(\begin{bmatrix} \boldsymbol{\alpha} \\ \mathbf{g} \end{bmatrix} |\sigma_e^2, \sigma_g^2, \mathbf{y}\right) &\approx \begin{bmatrix} \frac{2}{\sqrt{\frac{\sigma_e^2}{2}}}\mathbf{W}'\mathbf{M}^{[\infty]}\mathbf{W} & \frac{2}{\sqrt{\frac{\sigma_e^2}{2}}}\mathbf{W}'\mathbf{M}^{[\infty]}\mathbf{Z} \\ \frac{2}{\sqrt{\frac{\sigma_e^2}{2}}}\mathbf{Z}'\mathbf{M}^{[\infty]}\mathbf{W} & \frac{2}{\sqrt{\frac{\sigma_e^2}{2}}}\mathbf{Z}'\mathbf{M}^{[\infty]}\mathbf{Z}+\frac{1}{\sigma_g^2}\mathbf{K}^{-1} \end{bmatrix}^{-1} \\ &= \frac{\sqrt{\frac{\sigma_e^2}{2}}}{2} \begin{bmatrix} \mathbf{W}'\mathbf{M}^{[t]}\mathbf{W} & \mathbf{W}'\mathbf{M}^{[t]}\mathbf{Z} \\ \mathbf{Z}'\mathbf{M}^{[t]}\mathbf{W} & \mathbf{Z}'\mathbf{M}^{[t]}\mathbf{Z}+\omega\mathbf{K}^{-1} \end{bmatrix}^{-1}. \end{aligned} \tag{32}$$
